# The Heterocycle Isostere Explorer: A Computational Tool for the Discovery of Novel Aromatic Heterocyclic Isosteres

**DOI:** 10.1021/acs.jmedchem.5c03118

**Published:** 2026-02-11

**Authors:** Matthew T. O. Holland, Víctor Sebastián-Pérez, Anthony R. Bradley, Fernanda Duarte, Paul E. Brennan

**Affiliations:** † Centre for Medicines Discovery, 6396 University of Oxford, NDM Research Building, Old Road Campus, Oxford OX3 7FZ, U.K.; ‡ Chemistry Research Laboratory, University of Oxford, Mansfield Road, Oxford OX1 3TA, U.K.; § Exscientia Plc., The Schrödinger Building, Oxford Science Park, Oxford OX4 4GE, U.K.; ∥ Department of Chemistry, 4591 University of Liverpool, Crown Street, Liverpool L69 7ZD, U.K.; ⊥ Department of Computer Science, 4591 University of Liverpool, Brownlow Hill, Liverpool L69 7ZX, U.K.

## Abstract

Aromatic heterocycles are central to many areas of chemistry and play an important role in medicinal chemistry, where they are frequently used as bioisosteres to modulate molecular properties while preserving potency. Here, we report the heterocycle isostere explorer (HCIE), an easy-to-use, open-source Python tool for identifying heterocyclic bioisosteres from a custom-built virtual library of over 500,000 optionally functionalized aromatic heterocycles. HCIE employs a unique vector-based alignment and ranks candidates using the combined shape and electrostatic similarity. We describe its use in a series of medicinal chemistry case studies and propose novel 5,5-bicyclic molecules as bioisosteres of 2-pyridine, a common motif in FDA-approved drugs. By integrating 3D similarity metrics with physicochemical descriptors, HCIE provides medicinal chemists with a practical and extensible framework for early stage compound design while highlighting underexplored heterocycles whose predicted utility may motivate the development of new synthetic methodologies.

## Graphical abstract

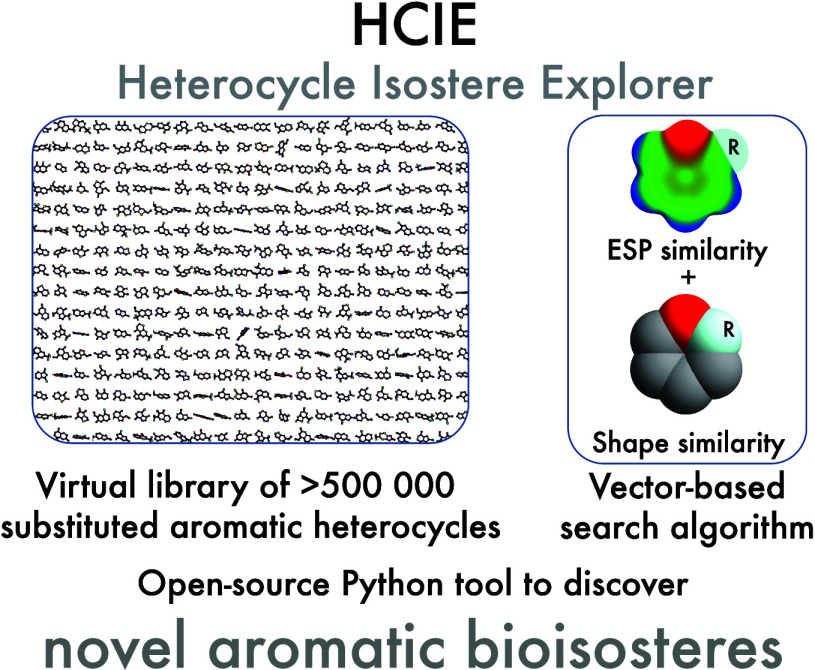

## Introduction

Aromatic heterocycles occupy a privileged region of chemical space, providing essential molecular functionality in diverse fields such as drug discovery, catalyst ligands, polymers, organic electronics, and photoswitches (Figure 1).^1^ Within medicinal chemistry, their importance has long been recognized, and their popularity is continuing to grow. Indeed, recent studies have found that 59% of small-molecule drugs approved by the U.S. Food and Drug Administration (FDA) between 1938 and 2012 contain nitrogen heterocycles, but in the 10 years between 2013 and 2023, this proportion increases significantly to 82%.^2–4^ These aromatic heterocycles frequently serve as core scaffolds in bioactive molecules, positioning key functional groups in 3D space to interact precisely with biological targets.^5^ Varying the heterocycle can thus impart useful alterations to the geometry of the molecule and its physicochemical properties, as well as allowing access to novel, patentable chemical space.^6^ Recently, azoheteroarenes, heteroarylhydrazones, and heterocyclic hemithioindigos have been shown to be effective photoswitches with uses in materials and supramolecular chemistry, where the identity of the heterocycle dictates both their photophysical properties and the lifetime of their photostationary states.^7–12^ Further applications in organoelectronics, in particular improving the efficiency of organic light-emitting diodes (OLEDs), polymers, and as ligands for highly selective catalysts of important organic transformations are continuing to be reported.^13–17^

**Figure 1:**
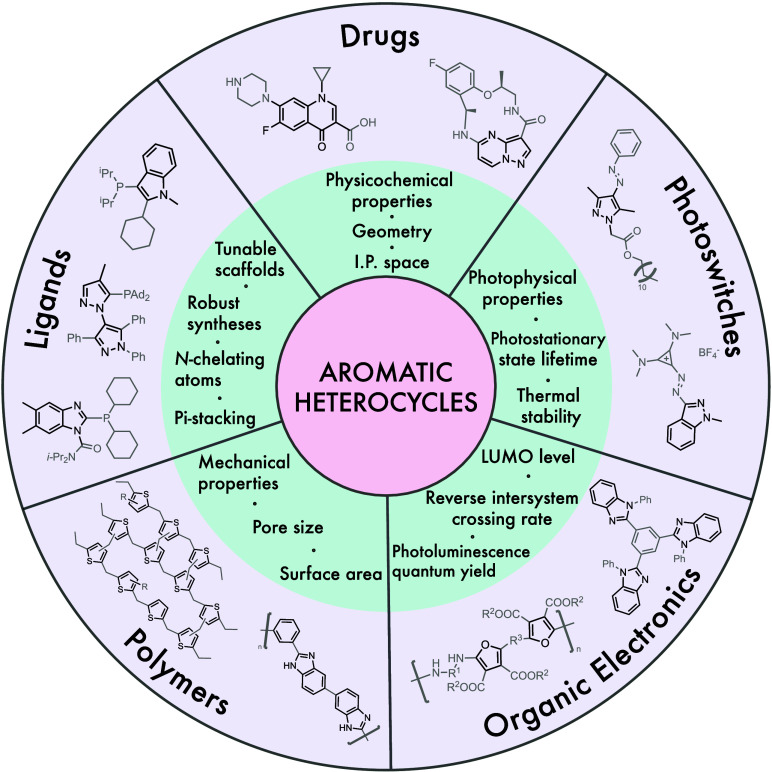
Some selected uses of aromatic heterocycles in chemistry, and the molecular properties affected by the heterocycle employed.^6,12–18^

Throughout all of these applications, the properties of the parent molecule depend strongly on the identity of the heterocycle, and thus exchanging the heterocyclic moiety is often found to be an efficient way of controlling these desired properties.

In medicinal chemistry, heterocyclic replacement has been a successful strategy in a large number of drug discovery campaigns. Scaffold-hopping, where the central core of a molecule is exchanged, often to improve the ADMET (absorption, distribution, metabolism, excretion, and toxicity) profile of a molecule by altering its physical properties, is a common practice.^5,6,19–21^ However, it is widely accepted that the proportion of heterocyclic chemical space that is synthetically accessible and sampled is limited; one study estimates that all approved drugs only contain in the region of 120 unique aryl scaffolds, and a recent investigation by Novartis found that over 99% of a library of chemically reasonable heterocyclic compounds generated by the authors did not appear in ChEMBL or PubChem and had thus most likely never been synthesized.^22,23^

Significant effort has been expended in this field to develop tools both for the systematic identification of aromatic heterocycles with similar properties (termed bioisosteres) and to enumerate regions of chemical space to promote the exploration of unsynthesized but potentially chemically useful molecules.^23–29^ Among these methodologies, SwissBioisostere is a freely accessible web-based tool that uses matched molecular pairs (MMPs) to search the ChEMBL database for previously made bioisosteric pairings of the user input molecule, and the results are then returned in order of the prevalence of recorded literature pairs.^28^ Although useful, this relies on previously characterized bioisosteric pairings within regions of chemical space that have already been accessed and thus is not able to propose totally novel bioisosteres. Researchers at Pfizer described their in-house database of small molecules enumerated using simple chemical stability and synthetic accessibility rules.^24,30^ This library covers a complete region of chemical space similar to that described by Pitt et al. in 2009 but does not include any substituents on the aromatic rings, and the code is not freely distributed or open-source.^22^ As aforementioned, Novartis published a database of four million ring systems with specific relevance to medicinal chemistry, and this database covers a very large region of unexplored cyclic and heteroaryl chemical space, but no tools for searching it were released, and its large size likely limits systematic searching to users with access to high-performance computing.^23^

A long-standing interest within our group in the design and application of novel aromatic heterocycles, and the lack of an open-source tool for searching through a complete enumeration of aromatic heterocyclic chemical space, motivated us to develop software that would both enable the discovery of new bioisosteres and inspire methodology development toward chemically useful heterocycles that have not yet been synthesized. Herein, we report the development of the heterocycle isostere explorer (HCIE), a Python-based tool that uses a unique vector-based ligand alignment method to search for molecules that are similar in molecular shape and electrostatic potential within a custom-built enumeration of aromatic heterocyclic chemical space.

As bioisosterism inherently affects physicochemical properties, HCIE also reports fragment-level molecular descriptors (molecular weight [MW], calculated log *P* [clog *P*], topological polar surface area [TPSA], hydrogen bond-donor/-acceptor counts [HBA/HBD], and heavy atom composition) for both the query and the proposed bioisosteres, together with their differences. These data enable medicinal chemists to assess the likely impact of each proposed bioisostere on the drug-relevant properties of the parent molecule at the point of design. The modular design of HCIE further allows users to incorporate additional physicochemical descriptors appropriate for their specific project needs.

Here, we describe and evaluate HCIE’s performance in projects relevant to medicinal chemistry, specifically in the retrospective analysis of inhibitor series for the NLRP3 inflammasome. We also propose a class of unsynthesized bicyclo[3.3.0] (herein referred to as 5,5-bicyclic) heterocycles that are predicted by our methodology to be useful as bioisosteres of commonly used heterocycles, with a range of physicochemical properties. HCIE is freely available as an open-source Python package distributed under a permissive MIT license for further applications within the scientific community.

## Results and Discussion

To retrieve potential isosteric compounds from the database, a query molecule needs to be (1) aligned effectively to each of the molecules in the database (herein referred to as probe molecules) and (2) each alignment needs to be scored according to appropriate criteria to determine similarity. Finally, the most similar compounds need to be returned to the user in a format where the most appropriate alignments and their scores are easy to visualize and further analyze. Ideally, this process should be quick enough that a user can run a search within 15 min, and the software tool must be straightforward to use for those with limited programming experience.

### Database Preparation

As a starting point, we took the virtual exploratory heterocyclic library (VEHICLe) published by Pitt et al. in 2009.^22^ This virtual database of 24,867 unsubstituted aromatic heterocycles represents a complete enumeration of the region of heterocyclic chemical space defined by the following rules:

- Mono- or bicyclic systems of five- or six-membered rings;
- only containing the elements {C, N, S, O, H};
- obey Hückel’s 4*n* + 2 rule of aromaticity; and
- exist only in the keto tautomer (only exocyclic carbonyls).

Annular tautomers were treated as distinct entries; thus, all annular tautomeric forms of each heterocycle are included as separate structures in VEHICLe. At the time of publishing, the authors found that only 1701 of the VEHICLe heterocycles had documented syntheses, and although this number is likely to have increased, the vast majority of these molecules remain unsynthesized.

To increase the chemical space accessible to HCIE, we expanded VEHICLe by adding substituents to each heterocycle, as illustrated in Figure 2. These were selected based on a 2017 analysis of molecules in the ChEMBL database and were amended based on our own experience of aromatic ring substitutions in medicinal chemistry.^31^ In choosing these, we sought to maximize the electronic diversity of the heterocycles in the database while minimizing the conformational flexibility of the substituted molecules. We selected separate sets of substituents depending on whether the attachment point on the heterocycle was an aromatic carbon (Figure 2A) or nitrogen (Figure 2B).

**Figure 2:**
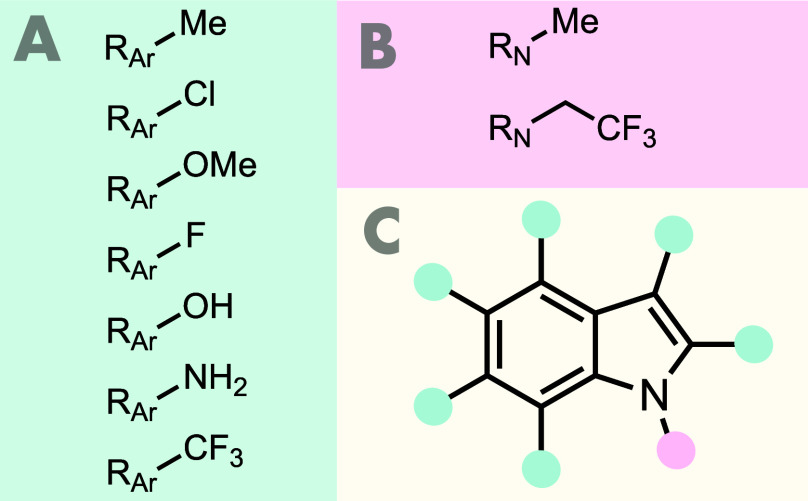
Functionalizing the VEHICLe database. (A) Substituents added to aromatic carbons. (B) Substituents added to “pyrrole-like” nitrogens. (C) The process of functionalizing indole as an example. Each C–H exit-vector (colored in green) is functionalized with each of the substituents in A in turn, giving 42 separate molecules. For the “pyrrole-like” nitrogen (colored in pink), each of the two substituents in B is added, giving a further 2 molecules. This will give a total of 45 molecules for the database: 44 monofunctionalized + unfunctionalized indole.

To functionalize the VEHICLe molecules, each C–H bond on an aromatic carbon and each N–H bond on an aromatic nitrogen was designated as a functionalizable bond (herein referred to as an exit-vector). We then generated a set of monofunctionalized heterocycles by systematically bonding each appropriate substituent to every possible exit-vector in each VEHICLe molecule, returning each functionalized molecule as a SMILES string.

This process was repeated for each of the monofunctionalized heterocycles to generate a further library of bifunctionalized heterocycles. The SMILES strings of the monofunctionalized and bifunctionalized molecules were then combined with the SMILES strings of the VEHICLe heterocycles to form the database. The total number of molecules, after symmetrically equivalent molecules were removed, is given in Table 1. Each heterocycle was then assigned a unique identification number (herein termed a RegID).

**Table 1:** Number of Heterocycles in Each of the Libraries after Monofunctionalizing and Bifunctionalizing with the Substituents Illustrated in Figure 2, and the Numbers Removed for Lack of Exit-Vectors or when Filtering for PEBs

library	heterocycles	without exit-vectors	PEBs	not flat	remaining
VEHICLe	24,867	1476	15,540	202	7649
monofunctionalized	336,816	24,746	172,471	4198	135,401
bifunctionalized	486,220	40,091	86,644	1567	357,918
total	847,903	66,313	274,655	5967	500,968

### Filtering

The method of library enumeration described above covers a complete section of aromatic heterocyclic chemical space and thus includes common motifs frequently encountered in synthetic chemistry (for example, benzene and pyridines) but also molecules that could never stably exist or be useful synthetically (due either to inherent instability or reactivity or by having no available exit-vectors for bonding to further functionality). While it was our desire to include as many as-yet unsynthesized molecules as possible (leaving it to the end user to decide what is or is not feasible), we wished to avoid including molecules that are chemically unstable or so reactive as to not be useful in any medicinal application. We therefore filtered the library using a minimal set of rules designed to remove these “potentially explosive or bonkers” (PEB) molecules. Molecules were thus removed from the library if they met one or more of the below rules:

- fewer than 20% of heavy atoms are carbon (tetrazole was manually excluded from this filter);
- contain more than three aromatic nitrogens bonded together (in a linear or branched arrangement);
- contain four or more heteroatoms bonded together (in a linear or branched arrangement);
- contain three or more exocyclic carbonyls;
- contain a cyclic acid anhydride;
- contain a cyclic thioester;
- contain two nitrogens bridging a fused ring system;
- contain two or more sulfurs within a six-membered ring; or
- contain two cyclic esters in which the carbonyl carbons are directly bonded.

Examples of molecules with these features, and the SMARTS expressions used to filter the data set, can be found in Section S-1 of the Supporting Information.

The heterocycles with no remaining exit-vectors were then identified and removed from the filtered database, as heterocycles that cannot be bonded to further functionality are effectively useless as bioisosteres. During the searching process, the heterocycles undergo geometry optimization including a final optimization using the MMFF94 force field (see Geometry and Partial Charges below). A small number of the remaining heterocycles (≈1%) adopted a puckered conformation after optimization (see Supporting Information Section S-1) indicative of a lack of aromatic character, and thus these were also removed from the database.

After filtering (see Table 1), a final database of 500,968 aromatic heterocycles remained, which we have termed MoBiVic (the Mono- and Bifunctionalized Vehicle database). The physicochemical property profiles of the MoBiVic heterocycles closely align with those of scaffolds commonly used in drug discovery and are thus directly useful within hit-to-lead optimization workflows (an overview of the physicochemical properties of MoBiVic and VEHICLe is shown in Section S-2 of the Supporting Information). MoBiVic is provided to the user as SMILES strings, and all of the libraries in Table 1 are freely distributed on GitHub. As the MoBiVic heterocycles are derived from VEHICLe, most of which are unsynthesized, we expect many of the MoBiVic structures to be novel and commercially unavailable.

We intentionally do not filter MoBiVic by synthetic accessibility because the feasibility of a given heterocycle depends strongly on its context within a parent molecule; particularly the electronic character of substituents, proximal functional groups, and the overall synthetic route. Any synthetic accessibility value calculated for the isolated heterocycle would, therefore, be of limited practical meaning. Nevertheless, to provide a very simple assessment of the success of the filtering rules described above, and to gauge whether the library is dominated by motifs that are far removed from drug-relevant chemistry, we evaluated the MoBiVic heterocycles using the Synthetic Bayesian Accessibility (SYBA) scoring metric.^32^ SYBA is a data-driven Bayesian metric that assesses the frequency of local atomic environments in large sets of previously synthesized versus contrived or highly complex molecules and therefore provides a statistical indication of whether the MoBiVic heterocycles resemble substructures commonly encountered in medicinal chemistry. With this intentionally simple measure, over 97% of MoBiVic heterocycles returned positive SYBA values (see Supporting Information Section S-2), indicating that the library is not enriched in intrinsically exotic or chemically implausible ring systems. We emphasize that this analysis does not predict synthetic routes or accessibility of complete compounds, which remain context- and project-dependent and must be assessed by the user using appropriate retrosynthetic or experimental tools. We anticipate that MoBiVic includes many synthetically accessible but as yet unexplored heterocycles with the potential to serve as potent bioisosteres and therefore propose it as a platform for expanding the scope of bioisosteric chemical space.

### Alignment and Scoring

Recognizing that the geometric arrangement of the exit-vectors in a heterocycle is critically important to its usefulness in medicinal chemistry, we implemented an alignment that is focused on minimizing the root mean squared displacement (rmsd) between the exit-vectors specified by the user and those in each of the MoBiVic molecules. This unique alignment method ensures both that the returned molecules are good isosteric matches and also that the proposed isosteres will project functionality at the correct geometric orientation relative to the query. As described below, the alignment methods differ based on whether a user specifies a single exit-vector or a pair of exit-vectors in their input.

### Geometry and Partial Charges

We wanted HCIE to be highly extensible, and as such we minimized the number of precalculated properties distributed with MoBiVic, and the external software dependencies, thus allowing users to alter the calculation methods if desired. As such, the geometries of the input heterocycle and each of the MoBiVic heterocycles are optimized from their SMILES strings at runtime, alongside the calculation of the partial charges used to approximate the electrostatic surface potential (ESP). With over 500,000 heterocycles in MoBiVic, the baseline methods for geometry optimization and partial charge calculation need to be computationally efficient to avoid adding a significant time overhead to each search.

Given the low degree of conformational flexibility in the MoBiVic heterocycles (the average number of rotatable bonds per molecule is 0.3) and the aforementioned need for efficiency, we generated a single low-energy conformer for the user input and each MoBiVic heterocycle with the ETKDG optimization algorithm as the baseline geometry method and partial charges calculated using the parametrized method described by Gasteiger in 1980.^33,34^ Both methods are efficiently implemented in the RDKit Python package, and in benchmarking studies (see Supporting Information Section S-3), these methods gave strongly correlated geometries and partial charges to those generated by higher level density functional theory (DFT) methods and the widely used restrained electrostatic potential (RESP) and Austin Model 1 bond charge correction (AM1-BCC) charges in a significantly shorter time (an average of 3.7 ms per heterocycle for ETKDG, vs 1 h 40 min for the DFT methods).

Although Gasteiger charges provide a simplified description of heterocycle electrostatics, we show them to be strongly correlated with those calculated by higher-level DFT methods, and to yield charges in a physically sensible range. More importantly for HCIE’s intended use, we explicitly assessed the stability of similarity rankings with respect to the choice of partial charge model. Validation of the HCIE search methodology against RESP and AM1-BCC charges by running a search for a case-study query, using a representative subset of 50 MoBiVic heterocycles as the searchable library, showed substantial agreement in the rank ordering of proposed bioisosteres, with both alternative charge models sharing 15 of the top 20 ranked molecules with the Gasteiger-based results and exhibiting Spearman rank correlation coefficients of 0.89 and 0.88 respectively (full details are given in Supporting Information Section S-3). These results demonstrate that HCIE’s prioritization of candidate bioisosteres is largely insensitive to the specific charge model employed, indicating that relative electrostatic patterns, rather than absolute charge values, dominate the ESP similarity scoring within HCIE.

Taken together, these results indicate that Gasteiger charges are sufficient for capturing relative electrostatic similarity trends and are, therefore, appropriate for use within the HCIE framework. HCIE is written in such a way that it is straightforward for users to run searches with higher-level charges to suit the requirements of individual projects.

We note that annular tautomers can present substantially different electrostatic surface potentials (ESPs) as the positions and protonation patterns of heteroatoms change the local charge distribution. To address this, MoBiVic explicitly includes annular tautomers as separate RegID entries (i.e., each tautomeric form is a distinct library member), so a HCIE search will return the tautomeric form in the library that is most similar in shape/ESP to the user’s query. For best results, the user should therefore provide the dominant tautomer/protonation state of their input fragment at the relevant experimental pH, or run HCIE on several likely protonation/tautomer forms and compare the results.

### Scoring

There are numerous published methods for quantifying the similarity between molecules.^35–38^ Inspired by Bolcato et al., we opted to score the query against each of the probes in the library using the Tanimoto shape similarity score (a similarity measure widely used in cheminformatics and implemented in RDKit) as the method of shape comparison, and the electrostatic potential similarity using a Python implementation of the method initially described by Good et al.^39,40^ This involves approximating the Coulombic potential of each molecule as a sum of three Gaussian functions and then analytically integrating these to determine the overlap of the charge fields on the probe and query molecules. ESPs are approximated by using atomic partial charges. The similarity is then calculated using the Tanimoto index, which quantifies the similarity between two molecules by calculating the ratio of the overlap between two molecular features (e.g., shape or ESP) to the total combined features of both molecules. In the case of volume overlap (used here as a metric for shape similarity), a query and probe molecule with the same volume and a perfect alignment would return a score of 1. Molecules that are not well aligned or have dramatically different shapes (and therefore have large regions of no overlap) would have a score closer to 0. For electrostatic similarity, if the regions of positive ESP line up well for a given alignment, the Tanimoto score will be high, and if the regions of positive ESP align with regions of negative ESP, the score will be low.

The overall similarity score for two molecules in a given alignment is found by summing directly the normalized shape similarity score and the electrostatic similarity scores. This then gives a score between 0 (least similar) and 2 (most similar). It is possible for the user to unequally weight the shape and ESP similarity scores in the calculation of the total score if required. This total score is intended as a pragmatic ranking metric. As a practical rule-of-thumb based on our experiences, total scores of ≈1.7 and ≈2.0 tend to correspond to very close isosteric matches, scores around ≈1.4 are generally good contenders for follow-up, and substantially lower scores are increasingly speculative. These thresholds should be treated only as approximate guides, as the precise significance of a given shape or ESP contribution is project-dependent, and we therefore recommend that users treat these values only as starting points.

### One-Vector Searching and Alignment

A query with a single user-specified exit-vector is aligned and scored against every probe in MoBiVic according to the procedure outlined in Figure 3. For each of the probes, all of the exit-vectors are enumerated, and these are sequentially aligned to the user-specified exit-vector in the query. These alignments are achieved using the Kabsch–Umeyama algorithm to find the rotation of the probe onto the query that minimizes the rmsd between the two sets of ring atoms (for further details, see Section S-4 of the Supporting Information).^41^ This alignment is then scored for both electrostatic and shape similarity according to the scoring method outlined above. To take into account the possible lack of symmetry in both the probe and the query, the probe is then rotated by 180° about the exit-vector and rescored. This process is repeated for each of the exit-vectors in the probe, and the alignment with the highest combined shape and electrostatic score is returned for each MoBiVic molecule.

**Figure 3:**
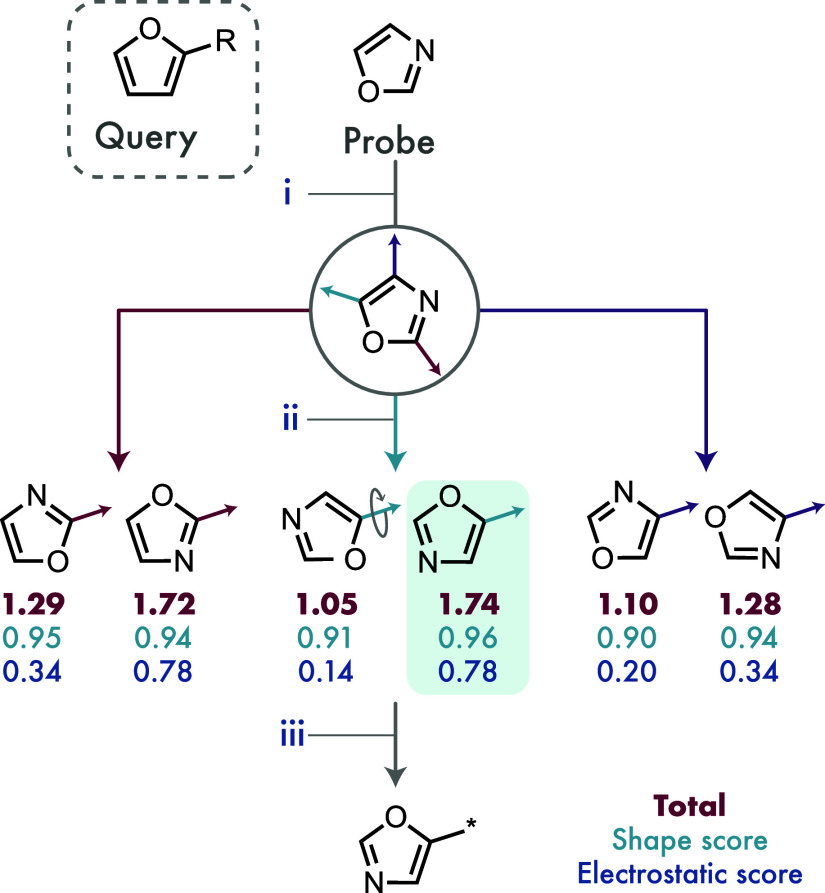
An illustration of the one-vector alignment process. Oxazole is used as an example to illustrate the alignment process, which is then repeated for every heterocycle in MoBiVic. (i) Each of the oxazole exit-vectors is identified (here indicated with colored arrows). (ii) Each oxazole exit-vector is aligned to the exit-vector on furan that was specified by the user, and the alignment scored for shape and electrostatic similarity. This alignment is rotated by 180° about the oxazole exit-vector and rescored, thus accounting for any asymmetry. (iii) The highest scoring alignment (here highlighted in green) for oxazole is then returned together with its scores. This process is repeated for all heterocycles in MoBiVic.

### Two-Vector Searching and Alignment

When two exit-vectors are specified in the query, the search process is different from that of the one-vector query. To ensure that only probe molecules with exit-vectors in the correct geometric arrangement are aligned and scored, an 8 bit binary label (hash) is first constructed that describes the geometry of the exit-vectors in the query molecule according to a method adapted from that proposed by Lauri and Bartlett in 1994.^42^ These hashes are precomputed for all possible pairs of exit-vectors in each MoBiVic molecule, and a lookup table is created, indexed by hash. This enables all probes with an orientation of exit-vectors similar to those in the query to be retrieved efficiently before aligning and scoring, thus reducing the time spent aligning to probes with an incompatible exit-vector geometry.

The hash takes into account the distance between the ring atoms of the exit-vectors and the angle between them, and its construction is outlined in Figure 4. The distance between the ring atoms of the exit-vectors (*d*) is calculated and assigned to one of 18 predefined bins (details of the designation of bin boundaries are given in Section S-5 of the Supporting Information), which are ordered by distance and numbered from smallest to largest. The angle between the two vectors (α_v_ as shown in Figure 4) is calculated and assigned to one of 6 bins in a similar manner. The bin numbers for the vector pair are then combined into an 8 bit binary hash, where the first five digits represent the distance *d* between the ring atoms, and the latter three digits the angle α_v_ between the vectors. Thus, two heterocycles that share the same hash both have at least one pair of exit-vectors with similar orientations and separation. As the bin boundaries have a degree of breadth, small geometric perturbations in the heterocyclic geometry are likely to fall in the same bins and thus yield identical hashes.

**Figure 4:**
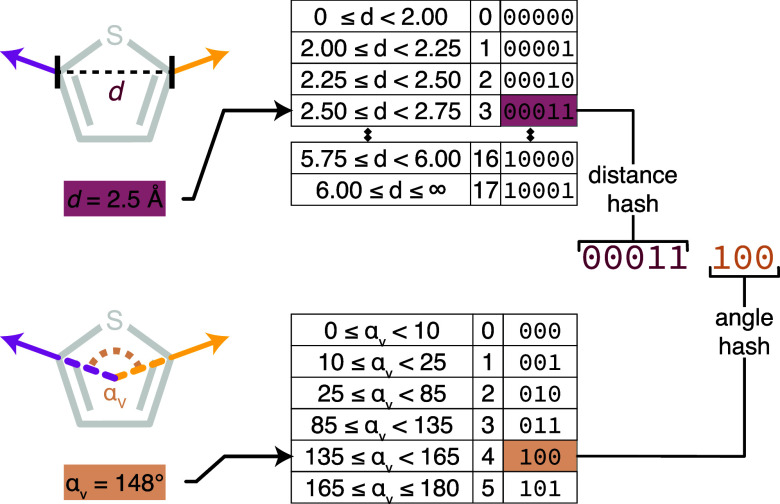
A demonstration of the process for calculating the hash for a 2,5-disubstituted thiophene query. The distance between the ring atoms of the two exit-vectors is measured and assigned to one of 18 bins (upper panel). The bins are ordered from smallest to largest distance and are each labeled with a 5 bit binary number. The angle between the C,N–H bonds of the two exit-vectors is measured and assigned to one of six bins (lower panel), again ordered from smallest to largest and labeled with a 3 bit binary number. The hash for the exit-vector pair is then constructed by appending the 3 bit angle label to the 5 bit distance label, thus forming an 8 bit hash that succinctly describes the geometry of the exit-vectors.

A two-vector search is illustrated in Figure 5. First, a hash is created for the user-defined exit-vectors in the query, and the RegIDs of the library heterocycles sharing this hash are then retrieved. This retrieval also includes mapping of the probe atoms that define the exit-vectors with the correct geometry. In a similar manner to that of the one-vector search, the exit-vectors of the correct geometry from each probe are aligned to those of the query using the Kabsch–Umeyama algorithm to minimize the rmsd between atoms in the exit-vectors. The alignment is then scored, and the exit-vectors in the probe are aligned to those of the query in the opposite order (akin to the 180° rotation in the one-vector alignment) and scored again. The highest scoring alignment is returned for each probe. This takes into account any asymmetry in the probe and ensures that the alignment of highest similarity is returned to the user.

**Figure 5:**
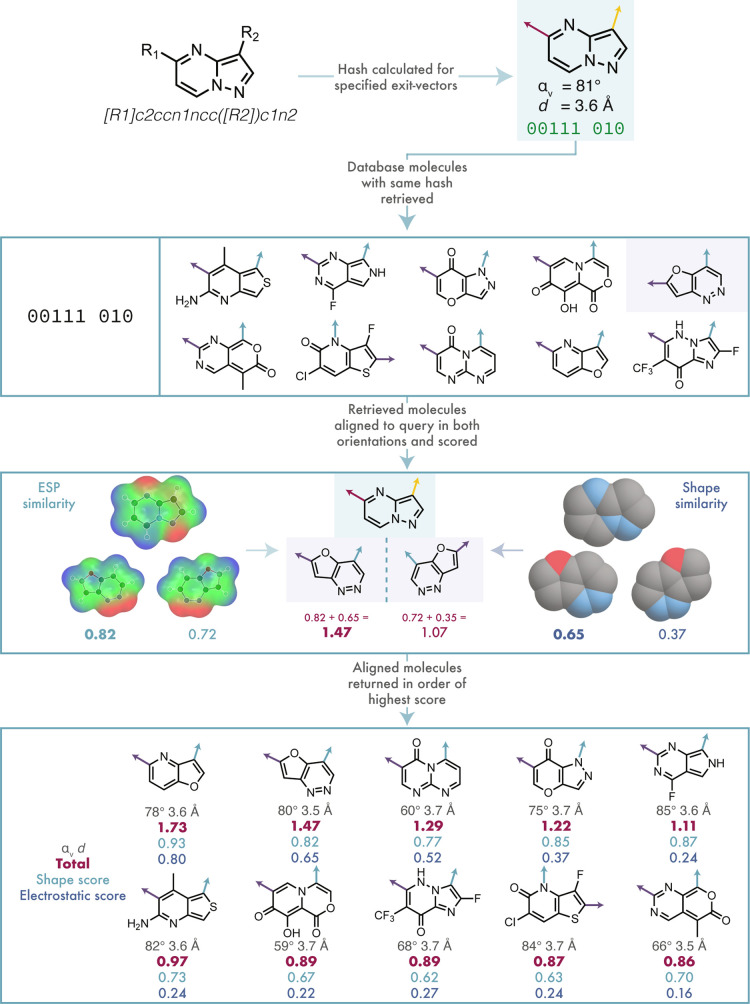
Method for searching and aligning molecules with two user-specified exit-vectors. The angles and distances between the exit-vectors in the example molecules are given to aid the reader in rationalizing the shape similarity scores.

### Output

A list of SMILES strings for all the probes that have been aligned and scored against the query is returned to the user as a *.csv* file ordered by total score, alongside the ESP and shape similarity scores, which is easily read into other programs for downstream analysis. In addition to the similarity scores, HCIE outputs fragment-level physicochemical properties for every returned heterocycle, thus allowing medicinal chemists to rapidly identify bioisosteric replacements that preserve shape and electrostatic complementarity while shifting physicochemical properties. A Structure Data File (SDF file) of the top 50 highest-scoring heterocycles in the alignment of the highest similarity is also returned, enabling users to view the geometries of the best alignments. To aid in rapid interpretation of the results, a *.png* file of the structures and scores of the top 50 heterocycles with the exit-vectors clearly defined is also returned to the user. As the code is open-source, users are free to adjust the outputs (including the number of returned heterocycles and the calculated physicochemical descriptors) to suit the preferences of their project.

### Runtime Scaling

To assess the runtime scaling of HCIE with database size, benchmarking on subsets of 10,000, 100,000, and the full MoBiVic library is provided in Supporting Information Section S-6, which demonstrated approximately linear scaling with database size.

### Case Studies

#### Bioisosteres for Pyrazolo­[1,5-*a*]­pyrimidine

3,5-Disubstituted pyrazolo­[1,5-*a*]­pyrimidine appears as a structural motif in the recent FDA-approved ROS1 tyrosine kinase inhibitor (ROS1TKI) repotrectinib, showing antitumor activity in non small cell lung cancers, including those with mutations that render them resistant to early generation therapies.^43^ This pyrazolo­[1,5-*a*]­pyrimidine also features in the first-in-class tropomyosin kinase inhibitor larotrectinib (approved by the FDA in 2018), where it is suggested to form a hydrogen bond with Met-592 in the hinge binding region, and is thus important for both potency and selectivity.^44,45^ Intrigued by the possibility of finding novel bioisosteres of this key motif, we performed a search on our database using the aforementioned methodology, which identified 95,071 molecules with the same exit-vector geometry, and aligned and scored each of these to the query in 129 s. The top 10 returned probes are shown in Figure 6, along with their respective scores.

**Figure 6:**
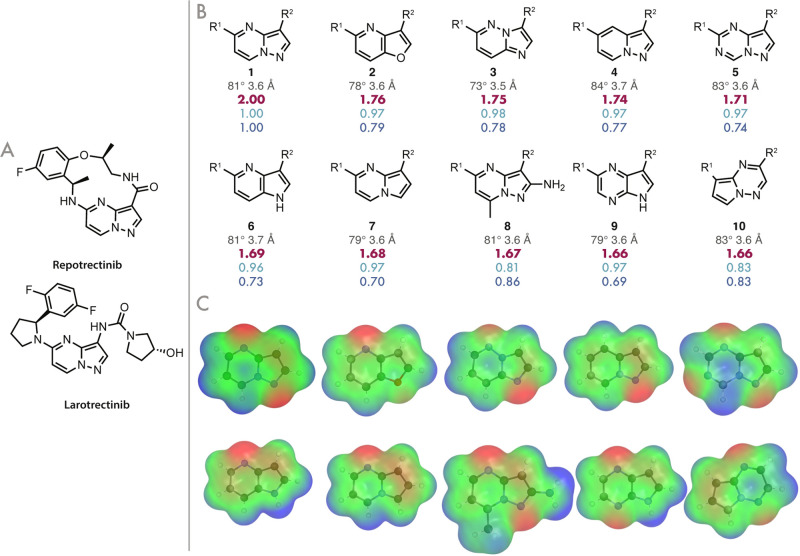
(A) The structures of repotrectinib and larotrectinib. (B) The top 10 returned molecules from a HCIE search of the bifunctionalized database, showing the total, shape, and ESP scores, and the angles and distances between their exit-vectors. (C) A visualization of the ESP surfaces for each of the heterocycles in (B). Regions of positive ESP are shown in blue, and regions of negative potential in red.

As expected, the query molecule was returned from the database with a perfect shape and an ESP score. Furthermore, all of the returned probes appear to be chemically reasonable, suggesting that the filters outlined above have succeeded in removing many of the less realistic molecules from MoBiVic. All but **6**, **7**, and **9** have an H-bond acceptor in the correct position to form the Met-592 interaction in the hinge binding region, and by inspection all of these have an exit-vector geometry corresponding to that of the 3,5-pyrazolo­[1,5-*a*]­pyrimidine query, showing the success of the hash-based searching in this context.

It is interesting to note that while the shape similarity scores are universally high across these top results, the ESP scores tend to be lower and more varied. The high shape scores are not surprising, as all of the returned scaffolds are 6,5-fused heterocycles and all bar **10** are aligned such that rings of the same size largely overlap in space. The lower shape similarity score of **8** can be attributed to the inclusion of the 2-amino-7-methyl substituents, which significantly increases the steric bulk about the central core when compared to that of **1**. The lower shape similarity of **10** is due to its returned alignment, with its 5-membered ring exit-vector aligned to the 6-membered ring exit-vector of the query, thus resulting in a poorer shape similarity than if the exit-vectors had been aligned to the query in the opposite manner. This demonstrates the balance to be struck between shape and ESP similarity when scaffold hopping, with the returned alignment having the second highest ESP similarity of all the returned non-query molecules (the exception being **8**, for which the scaffold is exactly the same, only decorated with electron-donating substituents). As the weightings of shape and ESP contributions to the total score were equal for this search, the increased ESP similarity that arose from the better aligning of the nitrogen–nitrogen bond in **10** with that of the query was significant enough to outweigh the penalty caused by the poorer spatial overlap. By inspection, it is not immediately obvious that this might be the case, and it is likely that **10** in the proposed alignment might be overlooked in a traditional, manually designed scaffold hop.

It has been established that, for certain targets, biological potency can depend sensitively on small differences in exit-vector orientation, with subtle changes in heteroatom placement leading to measurable differences in vector angles and, in turn, binding mode and selectivity.^46^ While the exit-vector angles and distances for the heterocycles shown in Figure 6 are closely clustered and do not themselves account for the observed differences in similarity scores, HCIE nevertheless provides access to sets of proposed bioisosteres that span both near-identical and subtly perturbed exit-vector geometries. This enables the systematic exploration of such geometric effects within SAR studies, alongside the orthogonal contributions of shape and electrostatic similarity.

The lower ESP scores can be explained by the sensitivity of the ESP to the precise arrangement of heteroatoms within the aromatic ring system.^6^ Different heteroatoms have different electronegativities, and so their precise arrangement within the ring has a significant effect on the overall electronic distribution, and thus influences the dipole moment and the angle between the exit-vectors. When comparing the ESPs of **6** and **9** to **1** (see Figure 6C), it is clear that introducing a “pyrrole-like” nitrogen (an H-bond donor) in place of the pyrazole’s “pyridine-like” nitrogen (an H-bond acceptor) results in a significant difference in ESP similarity, with negative potential about the 1-position of **6** and **9**, in contrast to the positive potential about the same position in **1**. Replacement of that nitrogen with a carbon as in **7** also has an impact on this similarity. It is interesting to note that the introduction of substituents has less of an effect on the ESP similarity scoring than changing the constitution of the heteroatoms, as **8**, which shares the same cyclic atoms as **1**, has the highest ESP similarity score to the query.

In addition to the ESP and shape similarities, the calculated physicochemical properties of the repotrectinib heterocyclic analogues (the parent molecule with the pyrazolo­[1,5-*a*]­pyrimidine substituted for the proposed heterocycle; summarized in Table 2) offer further insight into the suitability of the proposed heterocycles as bioisosteres. The changes in molecular weight, clog *P*, and TPSA are generally modest across these highest-scoring analogues, consistent with substitutions that preserve the balance of polarity and lipophilicity required for kinase hinge-binding motifs. Notably, the analogues constructed from heterocycles **4** and **7** reduce the TPSA relative to the pyrazolo­[1,5-*a*]­pyrimidine core, whereas **5** and **8** introduce modest increases in the polarity. Similarly, **2** and **6** offer small increases in lipophilicity with only modest changes in molecular weight, whereas **5** and **8** reduce the relative lipophilicity. Consistent with these trends, qualitative solubility predictions for the complete analogue structures (Table 2) indicate broadly comparable aqueous solubility classifications across the series, with no analogue predicted to exhibit an extreme loss of solubility relative to the parent scaffold. The relative advantage or disadvantage offered by these physicochemical adjustments depends on the optimization objective, but the inclusion of these fragment-level descriptors provides users with an immediate indication of how each proposed bioisostere will influence the drug-like properties of the parent molecule.

**Table 2:** Changes in the Calculated Physicochemical Properties of Repotrectinib Analogues Obtained by Substituting the Pyrazolo­[1,5-*a*]­pyrimidine Core with the Indicated Heterocycle^a^

heterocyclic analogue	ΔMW	Δclog *P*	ΔTPSA	SA score^b^	QED^b^	solubility^c^
original (**1**)	0	0.00	0.0	5.1	0.65	high
**2**	0	+1.10	–4.2	5.1	0.64	moderate
**3**	0	0.00	0.0	5.1	0.65	moderate
**4**	–1	+0.61	–12.9	5.1	0.65	high
**5**	+1	–0.60	+12.9	5.1	0.64	moderate
**6**	–1	+0.84	–1.5	5.0	0.58	moderate
**7**	–1	+0.61	–12.9	5.1	0.65	moderate
**8**	+29	–0.11	+26.0	5.2	0.55	moderate
**9**	0	+0.23	+11.4	5.1	0.58	high
**10**	0	0.00	0.0	5.0	0.65	high

a These are calculated as the sum of fragment contributions, thus the change for the analogue is the same as the contribution of the heterocyclic fragment.

b SA scores and quantitative estimate of drug-likeness (QED) scores were calculated for the analogues using RDKit, with default weightings for QED scores.

c Solubility category at pH = 7.4 predicted by ChemAxon.

To investigate the accessibility of these analogues, we calculated synthetic accessibility scores for each of them using the SA Score metric described by Ertl and Schuffenhauer and implemented in RDKit.^47^ The SA scores for each analogue are closely clustered across the proposed bioisosteres and are all very similar to the original molecule **1**, suggesting that these heterocyclic isosteres are of broadly comparable synthetic complexity to the parent pyrazolo­[1,5-*a*]­pyrimidine scaffold. In addition, to provide an initial assessment of potential metabolic liabilities, we evaluated predicted cytochrome P450 (CYP) 3A4 inhibitory activity for the complete analogue structures shown in Figure 6 using the CYPi-DNN predictor.^48^ None of the proposed analogues was predicted to inhibit CYP3A4, suggesting that the heterocyclic substitutions do not introduce an obvious CYP-mediated toxicity risk. Quantitative estimate of drug-likeness (QED) scores were also calculated for these analogues and show no significant variations across the series.^49^ Together, these calculations illustrate how HCIE outputs can be used as a starting point for user-defined downstream analyses, enabling proposed bioisosteres to be evaluated and prioritized within the context of project-specific synthetic or ADMET considerations.

Two interesting examples of molecules that would likely have been given equal prominence in a brute-force enumeration are **11** and **12**, as shown in Figure 7. These molecules appear visually similar to those in Figure 6 and would likely be proposed as candidates and considered in a manual, chemist-lead isostere search or a brute-force enumeration; however, their ESPs are very different from that of **1** (see Figure 7B) and are thus low-scoring by HCIE, suggesting that they would be poor bioisosteric replacements. These serve to highlight the utility of HCIE’s ESP and shape-based scoring for prioritizing isosteric candidates.

**Figure 7:**
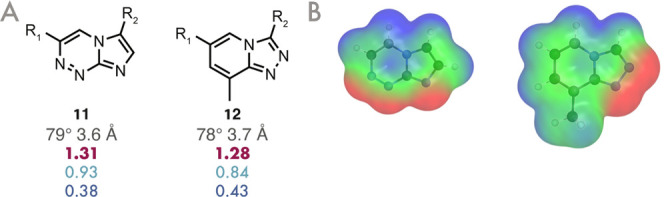
(A) Examples of visually plausible isosteric candidates that are scored poorly by HCIE. (B) Their ESP plots, demonstrating the significant differences in ESP compared to **1**.

To investigate whether any of these proposed heterocycles had previously been used as bioisosteres in scaffold-hopping studies, the 3,5-pyrazolo­[1,5-*a*]­pyrimidine query was searched in the SwissBioisostere database.^28^ 44 results were returned; thus, there exist 44 MMPs in the ChEMBL database where the query molecule has been directly substituted for another molecular motif (the full set of results are given in Supporting Information Section S-8). Of these molecular replacements, 14 would never appear in HCIE results, either as they are not included in our searchable database or the substitution pattern is not one included in our search methodology (for example, N-substitution on an amino group, or O-substitution on a phenol group). A further 7 of these did not have the same exit-vector geometry as the query (and thus have a different calculated hash) and so would not be included in the returned results. Pleasingly, **3** was the bioisostere with the highest frequency and best bioactivity improvement in the SwissBioisostere results, appearing in 33 MMPs of which 30 improved or retained bioactivity. **4** appeared as the sixth most frequent scaffold hop in the SwissBioisostere results with 5 MMPs, all of which retained or improved bioactivity. It is not surprising that **6** or **9** do not appear in the returned results as these lack a H-bond acceptor in the “pyrazole-like” nitrogen position as aforementioned. However, **2**, **4**, **5**, **7**, **8**, and **10** have not been used as bioisosteric scaffolds for 3,5-disubstituted pyrazolo­[1,5-*a*]­pyrimidine. Searching the chemical literature using Reaxys and SciFinder revealed that each of these ring systems has previously been synthesized, thus suggesting a lack of synthetic accessibility is unlikely to be the reason for these pairings never having previously been attempted. Furthermore, just because these pairings have never been made does not mean that these are not effective bioisosteres. These results thus demonstrate the utility of a vector-based alignment and scoring algorithm, paired with a sensibly curated virtual enumeration of chemical space, for the suggestion of novel aromatic heterocyclic bioisosteres.

#### Rationalizing the Activity of Inhibitors of the NLRP3 Inflammasome

In recent years, there has been significant interest in the role of inflammation in neurodegenerative disorders such as Alzheimer’s and Parkinson’s disease.^50–53^ The activation of the NLRP3 (NOD-, LRR-, and pyrin-domain containing protein 3) inflammasome has been implicated in Alzheimer’s Disease (AD), and there has been a recent surge in efforts to discover novel small-molecule inhibitors of this key part of the innate immune response for the treatment of neurodegenerative disorders and peripheral inflammation.^54–57^ A long-standing interest in small-molecule therapeutics for the treatment of neurodegeneration, coupled with an interest in the relative importance of shape and electrostatic complementarity in bioisosterism and bioactivity triggered by the results from our software described above, led us to investigate whether patterns in the potency of NLRP3 inhibitors could be rationalized by shape and electrostatic scoring.^58^

A data set of 8974 unique small-molecule inhibitors of the NLRP3 inflammasome, extracted from patent and published literature and annotated with IC_50_ data, was analyzed to find matched molecular series corresponding to aromatic heterocyclic replacements within our database. This was performed using the mmpdb Python package described by Dalke et al., where the ligands were fragmented using a SMARTS expression that prevented cuts being made on bonds to substituents that are included in our database (see Figure 2), and then the series were identified by grouping together all MMPs with the same constant fragment[Fn fn1].^59^ From these identified series were selected 5 that represented both single-vector and two-vector bioisosteric replacements, where the number of ligands in each series was large enough for the results to be meaningful, and the bioactivity data spanned a large range. The constant fragments defining these 5 series are shown in Figure 8, along with the number of ligands in each series. Three of these series (A–C) represent single-vector bioisosteric substitutions, with the remaining two representing two-vector scaffold hops. Series D is derived from CRID3, a commercially available NLRP3 inhibitor, and series E is a subset of series A where both the original constant fragment and an aromatic nitrile substituent are held constant.^60^

**Figure 8:**
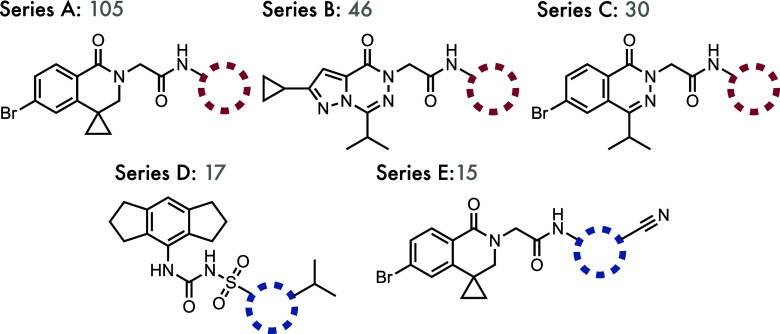
Structures of the scaffolds defining the five series of small-molecule NLRP3 inhibitors used in this study. The numbers by each series indicate the number of unique ligands in each series.

The variable fragment of the most potent ligand in each series was taken as the query, and all other variable fragments from the same series were aligned and scored against the relevant attachment vectors in the query using our software. Initially, the total score was calculated with equal weighting given to shape and ESP scores, and these were plotted against the pIC_50_ and Pearson correlation coefficients calculated (these data are shown in Section S-7 of the Supporting Information). The correlations between equally weighted total score and bioactivity gave positive scores in the range of 0.43–0.65, and all with a probability value below 0.05. As these scores are a much-simplified representation of the complex factors that determine binding affinity (for example, entropic contributions, macroconformational effects, or solvent interactions are not directly considered), a very high correlation coefficient would not be expected. Furthermore, correlation with experimentally derived binding affinities will always include a degree of experimental variability, which further reduces the expectation of a perfect correlation. These results demonstrate that there is a clear correlation between the shape and ESP similarities of aromatic heterocycles in these series and their binding affinity, thus supporting the hypothesis that the combined shape and ESP score are useful metric when considering isosteric replacements.

As with the data for 3,5-pyrazolo­[1,5-*a*]­pyrimidine above, for all series except E, the shape scores tend to be higher and with a more compact range than the ESP scores, which is likely to be for reasons similar to those described above.

Inspection of the distributions of the equally weighted total score and the constituent scores for each series (Supporting Information Section S-7), combined with the differences between shape and ESP similarities described for the bioisosteres of 3,5-pyrazolo­[1,5-*a*]­pyrimidine above, suggested differing contributions to the overall correlation between the two metrics, which might not be best represented in an equal weighting. For example, in series A, the shape scores correlate to binding affinity with a higher coefficient than that of the total score. To identify whether an unequal weighting in the total score would lead to better overall correlation with the binding affinity, a constrained optimization approach using the sequential least-squares programming (SLSQP) algorithm as implemented in SciPy was employed. The objective function was defined to minimize the negative Pearson correlation coefficient between the pIC_50_ and the weighted sum of the ESP and shape scores, with each weight constrained to the interval [0, 1], and the sum of weights equaling 1. The final weightings that led to the most positive correlation coefficient for each series are shown in Table 3.

**Table 3:** Optimized Weightings, as Calculated Using the SLSQP Optimization Algorithm for Each Series, and the Corresponding In-Series Pearson Correlation Coefficients for the Total Scores Calculated Using These Weightings

	weighting		Pearson coefficient	
series	shape	ESP	optimized	pre-optimization
A	0.90	0.10	0.55	0.43
B	0.22	0.78	0.61	0.58
C	1.00	0.00	0.72	0.58
D	0.93	0.07	0.76	0.62
E	0.41	0.59	0.66	0.65

As expected, many of these weightings deviate from the initial, equally weighted starting values, and correlating the weighted total score against pIC_50_ values leads to increases in Pearson correlation coefficients across all series, with the coefficients now lying in the range 0.55–0.76. It is interesting to note that there is not a consistent weighting across these series, with series A, C, and D showing a skewing toward shape similarity and series B and E toward electrostatic similarity. For series C, the optimum weighting takes no account of the ESP similarity, with the best correlated total score mirroring exactly the shape scores. These results suggest that the relative importance of steric and electrostatic complementarity in determining bioisosteres is target and ligand dependent, with different series being more “shape-like” or “ESP-like”. A retrospective optimization analysis such as this on existing data in a project enables the determination of the optimum weightings in calculating the total score for a given series, and these weights can be specified by the user when running subsequent searches with HCIE for prospective design.

This analysis rationalizes the pIC_50_ of the ligands in each series by assessing their similarity to the most potent in each series and shows that, although some series are better represented by their ligands’ shape or electrostatic similarity, there is no clear pattern to which will be a better representation. This suggests that, if data are available, a retrospective optimization analysis is likely to lead to better predictions of active bioisosteres than a simple equal weighting. However, the correlations between the equally weighted scores and the pIC_50_ are sufficiently robust to justify using equal weighting as a reasonable initial approach in the absence of prior data. Furthermore, this analysis was conducted using only ligands already synthesized and assayed as part of the discovery campaigns for each series, leaving open the possibility that more potent ligands that have yet to be designed or assayed exist in MoBiVic.

To assess whether the fitted weightings reflect genuine structure–activity trends rather than in-series overfitting, we performed repeated train/test splits within each series (see Supporting Information Section S-7). For the larger series (A,C), the relative contributions of shape and electrostatic similarity are stable across splits, and correlations between weighted similarity and bioactivity remain positive on held-out molecules. For the smaller series (D and E), held-out correlations exhibit greater variance, reflecting limited data. Taken together, these results indicate that the optimized weightings capture chemically meaningful, series-specific trends while reinforcing that equal weighting remains a reasonable default in the absence of sufficient project-specific data.

At the time of writing, the biological characterization of these bicyclic NLRP3 small-molecule inhibitors is mostly restricted to their cellular bioactivity; thus, it is likely their physicochemical or pharmacokinetic properties will need to be further optimized as more ADMET profiling is performed.^57^ Heterocyclic replacement is a well-known strategy for optimizing pharmacokinetic properties in lead-like molecules.^20,61,62^ As the relationship between shape and electrostatic similarity (as calculated with our methodology) and potency has been demonstrated, there is scope for our method to aid in the design of analogues that retain bioactivity while offering alternative physicochemical or pharmacokinetic properties, potentially expanding the chemical space for discovering superior bioisosteres.

#### A Novel Class of Bioisosteres for 2-Pyridine

A recent analysis by Marshall et al. found that the proportion of FDA-approved drugs containing a nitrogen heterocycle increased from 59% in the period spanning 1938–2012 to 82% between 2013 and 2023.^4^ In the latter period, pyridine supplanted piperidine as the most frequently occurring heterocycle in these small-molecule drugs, appearing in 54 of the 321 unique molecules approved in this period. The authors noted that 90% of these pyridines were substituted in the *ortho*(2)-position, thus making 2-pyridine the most frequently used aromatic heterocyclic moiety in approved drugs.

To investigate whether this methodology could propose interesting bioisosteres of this important motif in medicinal chemistry, 2-pyridine was searched through HCIE. The search took 11 min and 17 s and returned all 546,271 MoBiVic probes, aligned, scored, and ranked in order of highest to lowest, with 2-pyridine being returned as the top-ranked match (with a perfect score of 2.0). 2-Thiophene was returned as the highest scoring proposed bioisostere (with an equally weighted total score of 1.72). Thiophene is a known bioisostere of pyridine, and a search in SwissBioisostere revealed that the 2-pyridine to 2-thiophene substitution had been made 588 times in ChEMBL, improving or retaining bioactivity in 514 of these cases.

In order to determine the proportion of these returned results that were already known bioisosteres of 2-pyridine, enrichment factors were calculated using the results of a SwissBioisostere search for 2-pyridine as the “validated hits”. These known bioisosteres were then filtered to eliminate those that do not appear in our database and those for which the number of pairings that reduced bioactivity outnumbered those which improved or retained it. This left 389 known bioisosteres of pyridine. Enrichment factors were then calculated from the results of our software search by comparing the number of known bioisosteres in a top fraction of the ranked HCIE results to the number that would be expected if that fraction had been chosen at random from the database. These results are shown in Table 4.

**Table 4:** Enrichment Factors Calculated for 2-Pyridine by Comparing the Number of Known Bioisosteres (Extracted from SwissBioisostere) in the Top Fraction of Results to the Number That Would be Expected if That Fraction Were Selected at Random^a^

	HCIE		ElectroShape	
top *N* molecules	retrieved	EF	retrieved	EF
10	9	1264	5	702
25	15	843	17	954
50	23	646	23	646
100	31	435	23	323
10%	350	9.0	301	7.7
25%	376	3.9	324	3.3
50%	388	2.0	364	1.9
75%	389	1.3	381	1.3
100%	389	1.0	389	1.0

a An enrichment factor of 1 indicates a performance no better than random, and a factor larger than one indicates a better than random ranking of ligands.

The enrichment factors for the higher proportions of the HCIE results are large, showing that our methodology can retrieve known active bioisosteres significantly more effectively than a random draw. The large size of the searchable database is likely to enlarge these early enrichment factors, as the probability of drawing 9 active ligands at random from a database of over 500,000 molecules is very small; however, the proportion of known ligands in the top 10, 25, and 50 molecules is very encouraging. The top 10 returned molecules from the HCIE search (excluding the query 2-pyridine) returned 9 known bioisosteres from the SwissBioisostere results, with the top 25 returning 15, and the top 50 containing 23 known bioisosteres, all of which are known to improve or retain bioactivity. Additionally, a high-scoring molecule not found in the known bioisostere set does not imply that it is a false positive; rather, it suggests that it has not yet been tested or recognized as a bioisostere.

We also validated our vector-based alignment and scoring algorithm against an established and well-adopted technique. Ultrafast shape recognition (USR) algorithms are fast nonsuperpositional scoring methods that are widely used in virtual ligand screening, and the ElectroShape method described by Stuart Armstrong et al. incorporates electrostatic information as a fourth dimension in the precomputed descriptor for each molecule, thus comparing ligands by shape and electrostatic similarity.^63^ Using the ElectroShape implementation provided in the Open Drug Discovery Toolkit, descriptors were calculated for every molecule in MoBiVic, and their similarities calculated to those of pyridine.^64^ The ElectroShape calculation took the same amount of time as HCIE, and the results (ranked in order of highest similarity) were used to calculate enrichment factors as above, which are displayed in Table 4. That the number of retrieved molecules is the same or greater for the HCIE search (with the exception of the enrichments in the top 25 molecules, where ElectroShape retrieves 2 additional bioisosteres) demonstrates that our vector-based scoring method is a valid way of searching MoBiVic. Additionally, the HCIE search also returns the exit-vector alignment of the highest similarity, aiding compound design by directly matching MMP substituents onto new heterocycle cores.

To explore the possibility of discovering new bioisosteres within these results, the top 50 highest scoring molecules that did not appear in the SwissBioisostere results were examined. Among these, we were interested to observe that a number of unusual 5,5-bicyclic molecules scored highly for similarity to the 2-pyridine query. 5,5-Bicyclic aromatic heterocycles are seldom encountered in medicinal chemistry; however, examples of their inclusion in antimicrobial candidates and enzyme inhibitors exist in the literature, suggesting that this large class of heterocycles contains untapped biological potential (it is interesting to note that Meanwell and Sistla have made comparable observations about the structurally similar pyrroloazoles).^65–68^ These molecules (which we propose as interesting new bioisosteres of 2-pyridine), their scores, and their physicochemical properties relative to 2-pyridine are shown in Figure 9. We emphasize that these 5,5-bicyclic heterocycles are proposed as hypotheses for bioisosteric replacement rather than as immediately deployable motifs. Their synthetic accessibility and suitability will ultimately depend on substitution pattern and molecular context, and must be evaluated on a case-by-case basis.

**Figure 9:**
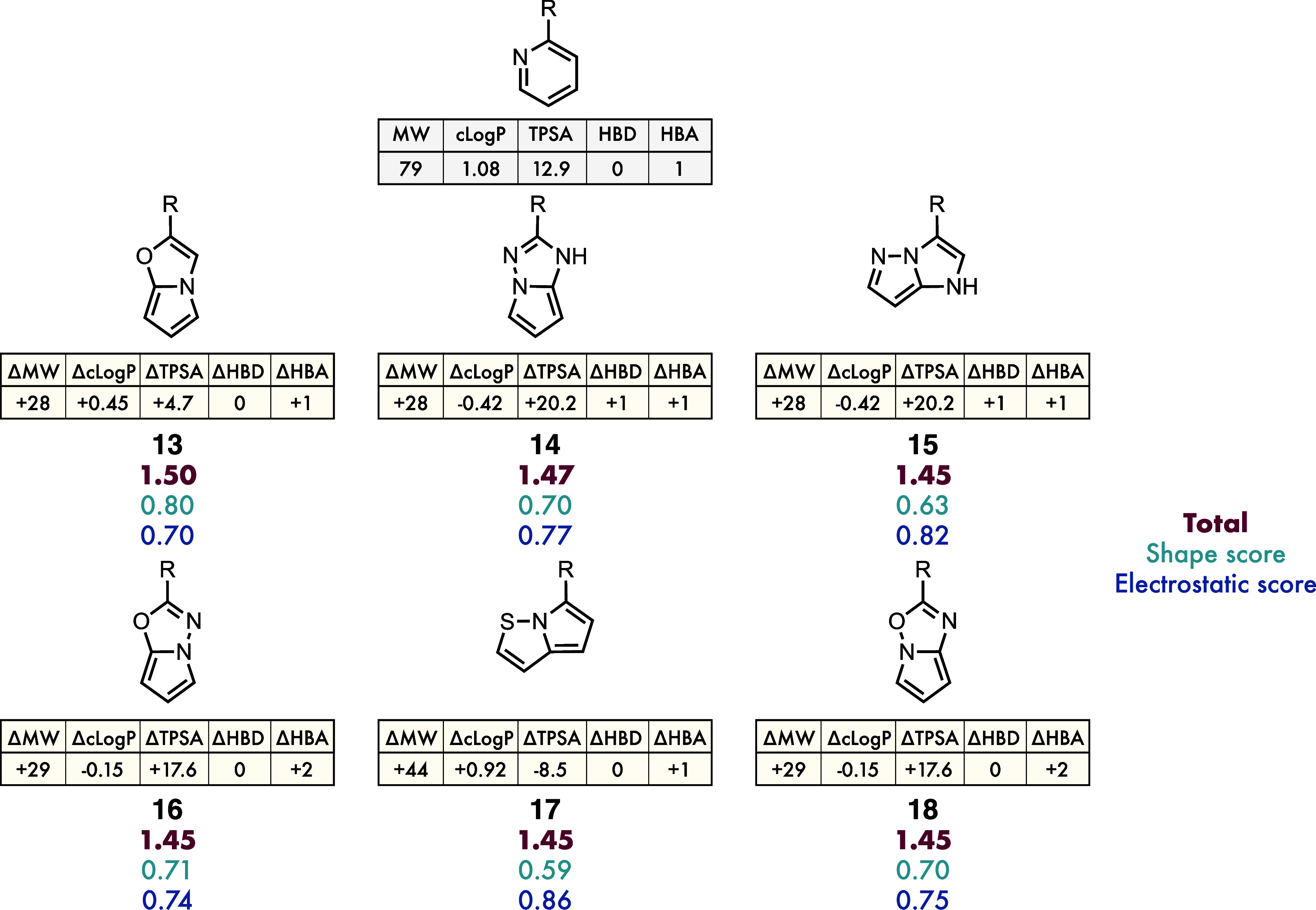
5,5-Bicyclic proposed bioisosteres of 2-pyridine, their scores, and their calculated physicochemical properties. The alignments represent those returned as the highest scoring alignment.

The physicochemical property changes accompanying these proposed substitutions provide insight into their potential utility as bioisosteres. As summarized in Figure 9, the six highlighted heterocycles span a useful range of increases and decreases in lipophilicity, TPSA, and hydrogen-bonding capacity. Such variations may be advantageous in different optimization contexts, for example, lowering TPSA or increasing clog *P* to improve permeability, or increasing polarity to modulate solubility or reduce metabolic liability. Together, these provide users with a toolkit of novel heterocyclic motifs that are predicted to retain the 3D interaction potential of 2-pyridine while offering access to distinct regions of physicochemical space, enabling substitution choices tailored to the specific optimization objectives of a given project.

The alignments of all of these molecules except **15** and **17** are unsurprising, with the long axis of the 5,5-bicycles aligned along the axis of the exit-vector in 2-pyridine. Interestingly, **15** and **17** are aligned with their axes skewed relative to that defined by the 2-pyridine exit-vector. This allows the ring-junction nitrogen in both cases to align with pyridine nitrogen of the query, giving large ESP similarity values that outweigh the effect on shape similarity caused by this skewed alignment. Whether this is an acceptable trade-off will depend on the precise nature of the binding pocket in the target.

Of these molecules, only **14** and **15** had previously been reported with the given substitution pattern (**14** appearing in the Markush structures of two separate patents), and **15** having a route reported in 1999 (but no reported inclusion in bioactive molecules until 2024).^69–72^ Until the beginning of 2024, there were no reported syntheses in the published literature for any of the remaining molecules. Pleasingly, in early 2024, a molybdenum-catalyzed deoxygenative coupling strategy to access various heteroaromatic scaffolds, including **13**, was reported by Wang et al.^73^ These previous reported syntheses suggest that members of this biologically under-explored class are synthetically accessible. It is hoped that, in light of their potential utility as bioisosteres of 2-pyridine and thus biological relevance, research efforts in the synthetic community can be streamlined toward developing further syntheses of these molecules and efforts put toward their inclusion and characterization in bioactive compounds. In our laboratory, we have been exploring routes toward accessing the structures in Figure 9. Our preliminary results support the chemical tractability of several of these scaffolds and will be reported separately.

### Limitations and Opportunities for Future Development

We believe that the HCIE methodology described here represents an efficient process for systematically screening aromatic heterocyclic chemical space for novel and useful isosteres. As with all computational methodologies, certain assumptions and simplifications have been made in the interests of efficiency and pragmatism in the implementation. Although these are consistent with other ligand-based virtual screening tools, it is important to acknowledge and appreciate these underlying assumptions when interpreting HCIE’s results.

Gasteiger partial charges were used to approximate the ESP around each molecule for the purposes of similarity scoring. Although the benchmarking outlined in Section S-3 of the Supporting Information demonstrates that these charges are appropriate given the computational efficiency with which they can be calculated, higher-level methods of calculating partial charges for representing molecular ESPs do exist (for example, restrained electrostatic potential charges; RESPs).^74^ Recognizing that there might be applications where higher-level charges are required, HCIE supports the use of externally calculated charges for determining ESP similarities.

Synthetic feasibility and other context-dependent properties represent important limitations of the present work. HCIE does not attempt to predict the synthetic accessibility, solubility, or global toxicity of heterocycles in MoBiVic in isolation, because these properties are strongly dependent on the full molecular context rather than the heterocyclic fragment alone. Although computational metrics exist for synthetic accessibility and ADMET end points, these are typically developed for complete molecules rather than isolated fragments.^47,48,75,76^ Applying these to isolated heterocycles in a project-agnostic manner, without taking into account the molecular context of the parent compound, is likely to be misleading and could hinder rather than clarify early stage design decisions. For this reason, we chose not to embed such metrics into HCIE. Assessment of synthetic tractability, solubility, and toxicity is intentionally left to the user, who is best placed to evaluate these attributes upon computational incorporation of HCIE output into complete molecules.

The HCIE methodology assumes that the query and library heterocycles can be considered as fragments isolated from the steric and electronic environment of a larger parent molecule. This is a common first-order approximation in ligand-based screening protocols, enabling the efficient exploration of chemical space. To assess the validity of this assumption in the context of HCIE, we performed a test based on the repotrectinib case study described earlier (and described in Section S-10 of the Supporting Information). We selected repotrectinib as the pyrazolo­[1,5-*a*]­pyrimidine heterocycle is bonded to both electron-donating and -withdrawing substituents. Repotrectinib analogues were generated in silico by replacing the pyrazolo­[1,5-*a*]­pyrimidine moiety with each of the 95,071 heterocycles with similar exit-vector geometries returned in the original search. The geometries of these analogues were optimized and the partial charges calculated using the same levels of theory as for a HCIE search. The heterocycles were then extracted from the parent molecule and re-searched as a library, this time using the geometries and charges calculated in the context of the analogue molecule. Pleasingly, eight of the top ten molecules illustrated in Figure 6 were present among the top ten highest scores, and 618 of the top 1000 were retrieved. While this demonstrates that the fragment-based approximation captures much of the relevant isosteric information, it also highlights that whole-molecule electronic effects should be considered in cases in which the heterocycle interacts strongly with distal parts of the parent molecule. Importantly, HCIE’s implementation allows users to provide geometries and charges calculated in the context of their own parent molecules, thus tailoring screening to specific contexts if required.

Our implementation of HCIE intentionally focused on ESP and shape similarity, which we view as the most broadly transferable and computationally efficient descriptors for exploring aromatic heterocyclic bioisosterism. We recognize that these two parameters do not capture every factor relevant to medicinal chemistry design. Fragment-level physicochemical descriptors are therefore included within HCIE to provide immediate insight into how each proposed bioisostere may influence the drug-like properties of the parent molecule. Other parameters (for example, p*K*_a_ or HOMO–LUMO gaps) can also contribute to bioisosterism, although the significance of these is often more context-dependent than ESP and shape similarity. The modular design of HCIE readily supports the integration of such additional metrics into the scoring process, either via calculation at runtime or precomputing values for the entire library. Likewise, the simple additive scoring framework allows users to apply project-specific weightings, ensuring that HCIE can be tailored to the requirements of diverse medicinal chemistry programmes.

## Conclusions

Herein, we have reported the development of a novel, vector-based method for aligning mono- and bisubstituted aromatic heterocycles, scoring these returned alignments based on shape and electrostatic similarity, and its implementation into a free and open-source Python package, the Heterocycle Isostere Explorer (HCIE). We also report the development of a virtual library of mono- and bisubstituted aromatic heterocycles (MoBiVic), covering a well-defined region of aromatic heterocyclic chemical space and incorporating simple filters to remove reactive or chemically unstable motifs. MoBiVic comprises VEHICLe-derived five- and six-membered monocyclic and fused bicyclic aromatic heterocycles containing only C, N, O, S, and H atoms. Applying HCIE to this library, we identified potential new bioisosteres for 3,5-disubstituted pyrazolo­[1,5-*a*]­pyrimidine, a motif found in several FDA-approved drugs. The activity of five series of small-molecule inhibitors of the NLRP3 inflammasome was rationalized based on the relative importance of shape or electrostatic similarity in each series, and corresponding weightings were derived to enable the prediction of new aromatic bioisosteres within these series. Finally, HCIE identified a series of largely unsynthesized 5,5-bicyclic scaffolds as promising bioisosteres for the medicinally important 2-pyridine motif.

Together, these case studies demonstrate that HCIE provides a practical and versatile tool for medicinal chemists, enabling the systematic identification of novel heterocyclic bioisosteres while offering access to alternative regions of the physicochemical property space. Importantly, the HCIE is designed to operate at the design stage, where geometric and electronic compatibility can be assessed independently of route-specific synthesis, solubility, or toxicity considerations that must ultimately be evaluated in the context of the complete molecule and the requirements of a given project. The MoBiVic library, coupled with HCIE’s alignment and scoring framework, therefore offers not only a means of prioritizing viable bioisosteric analogues but also a way of highlighting under-explored heterocyclic motifs whose predicted biological relevance may justify and help direct future synthetic efforts. In this way, HCIE provides both immediate support for medicinal chemistry optimization and a foundation for expanding the reach of synthetic heterocyclic chemistry into new and potentially bioactive regions of chemical space.

## Supplementary Material



## Data Availability

All calculations described were run on an Apple MacBook Pro equipped with an Apple M1 Pro chip, an 8-core CPU, and 16 GB of unified memory. The operating system was macOS 12.x. The source code and user-guide for HCIE is available at https://github.com/BrennanGroup/HCIE, and the MoBiVic database is available (as a collection of SMILES strings) at https://github.com/BrennanGroup/MoBiVic.

## Footnotes

a In MMP analysis, for a given matched molecular series, the parts of the molecule that remain the same are termed the constant, and the parts that change the variable.

## Notes

Details of the MoBiVic filtering, physicochemical properties of the MoBiVic database, benchmarking of the geometry optimization and partial charge calculations, explanation of the implementation of the Kabsch–Umeyama algorithm for heterocyclic alignment, determination of the bin boundaries for generating hashes, details of the runtime scaling benchmark tests, correlations for the NLRP3-matched molecular series, results of a SwissBioisostere search for 3,5-disubstituted pyrazolo­[1,5-*a*]­pyrimidine, VEHICLe sample used for geometry rmsd benchmarking, and explanation of the method used for molecular context benchmarking (PDF)

## #

SandboxAQ, 780 High Street, Palo Alto, CA 94301, USA

## Abbreviations

EF: enrichment factor
ESP: electrostatic surface potential
ETKDG: Experimental-Torsion Knowledge Distance Geometry algorithm
MMP: matched molecular pair
MMFF94: Merck Molecular Force Field 94
MoBiVic: Mono- and Bifunctionalized Vehicle database
NLRP3: NOD-, LRR-, and pyrin-domain containing protein 3 inflammasome
OLED: organic light-emitting diode
PEB: potentially explosive or bonkers molecules
RegID: registration identifier (unique heterocycle ID)
RESP: restrained electrostatic potential
ROS1: ROS proto-oncogene 1 receptor tyrosine kinase
SDF: structure-data file
SLSQP: sequential least-squares programming
TKI: tyrosine kinase inhibitor
USR: ultrafast shape recognition
VEHICLe: virtual exploratory heterocyclic library

## References

[1] Cabrele C., Reiser O. (2016). The modern face of synthetic heterocyclic chemistry. J. Org. Chem..

[2] Kerru N., Gummidi L., Maddila S., Gangu K. K., Jonnalagadda S. B. (2020). A review
on recent advances in nitrogen-containing molecules and their biological
applications. Molecules.

[3] de
la Torre B. G., Albericio F. (2024). The pharmaceutical industry in 2023:
an analysis of FDA drug approvals from the perspective of molecules. Molecules.

[4] Marshall C. M., Federice J. G., Bell C. N., Cox P. B., Njardarson J. T. (2024). An update
on the nitrogen heterocycle compositions and properties of U.S. FDA-approved
pharmaceuticals (2013–2023). J. Med.
Chem..

[5] Callis T. B., Garrett T. R., Montgomery A. P., Danon J. J., Kassiou M. (2022). Recent scaffold
hopping applications in central nervous system drug discovery. J. Med. Chem..

[6] Meanwell, N. In Adv. Heterocycl. Chem.; Scriven, E. F. , Ramsden, C. A. , Eds.; Academic Press, 2017; Vol. 123, pp 245–361.

[7] Crespi S., Simeth N. A., König B. (2019). Heteroaryl
azo dyes as molecular
photoswitches. Nat. Rev. Chem..

[8] Josef V., Hampel F., Dube H. (2022). Heterocyclic hemithioindigos:
highly
advantageous properties as molecular photoswitches. Angew. Chem. Int. Ed..

[9] Mukherjee A., Seyfried M. D., Ravoo B. J. (2023). Azoheteroarene and diazocine molecular
photoswitches: self-assembly, responsive materials and photopharmacology. Angew. Chem. Int. Ed..

[10] Hegedüsová L., Blaise N., Pašteka L. F., Budzák v., Medved M., Filo J., Mravec B., Slavov C., Wachtveitl J., Grabarz A. M., Cigáň M. (2024). Enhancing
the potential of fused heterocycle-based triarylhydrazone photoswitches. Chem.—Eur. J..

[11] Gernet A., El Rhaz A., Jean L. (2023). Easily accessible substituted heterocyclic
hemithioindigos as bistable molecular photoswitches. Chem.—Eur. J..

[12] Fink M., Stäuble J., Weisgerber M., Carreira E. M. (2024). Aryl azocyclopropeniums:
minimalist, visible-light photoswitches. J.
Am. Chem. Soc..

[13] Bangsund J. S., Fielitz T. R., Steiner T. J., Shi K., Van Sambeek J. R., Clark C. P., Holmes R. J. (2019). Formation
of aligned periodic patterns
during the crystallization of organic semiconductor thin films. Nat. Mater..

[14] Fu W., Kong L., Shi J., Tong B., Cai Z., Zhi J., Dong Y. (2019). Synthesis
of poly­(amine–furan–arylene)­s
through a one-pot catalyst-free in situ cyclopolymerization of diisocyanide,
dialkylacetylene dicarboxylates, and dialdehyde. Macromolecules.

[15] Luo Y., Li B., Wang W., Wu K., Tan B. (2012). Hypercrosslinked aromatic
heterocyclic microporous polymers: a new class of highly selective
CO2 capturing materials. Adv. Mater..

[16] So C. M., Yuen O. Y., Ng S. S., Chen Z. (2021). General chemoselective
Suzuki–Miyaura coupling of polyhalogenated aryl triflates enabled
by an alkyl-heteroaryl-based phosphine ligand. ACS Catal..

[17] Choi K., Brunn J. N., Borate K., Kaduskar R., Lizandara
Pueyo C., Shinde H., Goetz R., Hartwig J. F. (2024). Palladium-catalyzed
amination of aryl halides with aqueous ammonia and hydroxide base
enabled by ligand development. J. Am. Chem.
Soc..

[18] Coelho P. J., Castro M. C. R., Fernandes S. S., Fonseca A. M. C., Raposo M. M. M. (2012). Enhancement
of the photochromic switching speed of bithiophene azo dyes. Tetrahedron Lett..

[19] Meanwell N. A. (2011). Synopsis
of some recent tactical application of bioisosteres in drug design. J. Med. Chem..

[20] Jampilek J. (2019). Heterocycles
in medicinal chemistry. Molecules.

[21] Meanwell N. A. (2023). Applications
of bioisosteres in the design of biologically active compounds. J. Agric. Food Chem..

[22] Pitt W. R., Parry D. M., Perry B. G., Groom C. R. (2009). Heteroaromatic rings
of the future. J. Med. Chem..

[23] Ertl P. (2024). Database of
4 million medicinal chemistry-relevant ring systems. J. Chem. Inf. Model..

[24] Tu M., Rai B. K., Mathiowetz A. M., Didiuk M., Pfefferkorn J. A., Guzman-Perez A., Benbow J., Guimarães C. R. W., Mente S., Hayward M. M., Liras S. (2012). Exploring aromatic
chemical space with NEAT: novel and electronically equivalent aromatic
template. J. Chem. Inf. Model..

[25] Vainio M. J., Kogej T., Raubacher F., Sadowski J. (2013). Scaffold hopping by
fragment replacement. J. Chem. Inf. Model..

[26] Ertl P. (2014). Intuitive
ordering of scaffolds and scaffold similarity searching using scaffold
keys. J. Chem. Inf. Model..

[27] Lešnik S., Škrlj B., Eržen N., Bren U., Gobec S., Konc J., Janežič D. (2017). BoBER: web interface
to the base of bioisosterically exchangeable replacements. J. Cheminf..

[28] Cuozzo A., Antoine D., Marta A. S. P., Olivier M., Vincent Z. (2022). SwissBioisostere
2021: updated structural, bioactivity and physicochemical data delivered
by a reshaped web interface. Nucleic Acids Res..

[29] Ertl P., Altmann E., Racine S., Lewis R. (2022). Ring replacement
recommender:
Ring modifications for improving biological activity. Eur. J. Med. Chem..

[30] Fink T., Reymond J.-L. (2007). Virtual exploration
of the chemical universe up to
11 atoms of C, N, O, F: assembly of 26.4 million structures (110.9
million stereoisomers) and analysis for new ring systems, stereochemistry,
physicochemical properties, compound classes, and drug discovery. J. Chem. Inf. Model..

[31] Hall R. J., Murray C. W., Verdonk M. L. (2017). The Fragment
Network: A chemistry
recommendation engine built using a graph database. J. Med. Chem..

[32] Voršilák M., Kolář M., Čmelo I., Svozil D. (2020). SYBA: Bayesian estimation
of synthetic accessibility of organic compounds. J. Cheminf..

[33] Riniker S., Landrum G. A. (2015). Better informed
distance geometry: Using what we know
to improve conformation generation. J. Chem.
Inf. Model..

[34] Gasteiger J., Marsili M. (1980). Iterative partial equalization
of orbital electronegativity-a
rapid access to atomic charges. Tetrahedron.

[35] Bajusz D., Rácz A., Héberger K. (2015). Why is Tanimoto index an appropriate
choice for fingerprint-based similarity calculations?. J. Cheminf..

[36] Jiang Z., Xu J., Yan A., Wang L. (2021). A comprehensive comparative assessment
of 3D molecular similarity tools in ligand-based virtual screening. Brief. Bioinform..

[37] Shin W.-H., Zhu X., Bures M. G., Kihara D. (2015). Three-dimensional compound comparison
methods and their application in drug discovery. Molecules.

[38] Maia E. H. B., Assis L. C., de Oliveira T. A., da Silva A. M., Taranto A. G. (2020). Structure-based
virtual screening: From classical to artificial intelligence. Front. Chem..

[39] Bolcato G., Heid E., Boström J. (2022). On the value
of using 3D shape and
electrostatic similarities in deep generative methods. J. Chem. Inf. Model..

[40] Good A. C., Hodgkin E. E., Richards W. G. (1992). Utilization of Gaussian functions
for the rapid evaluation of molecular similarity. J. Chem. Inf. Comput. Sci..

[41] Kabsch W. (1976). A solution
for the best rotation to relate two sets of vectors. Acta Crystallogr. A.

[42] Lauri G., Bartlett P. A. (1994). CAVEAT: a program
to facilitate the design of organic
molecules. J. Comput. Aided Mol. Des..

[43] Drilon A. (2024). Repotrectinib in ROS1 fusion-positive non-small-cell lung cancer. N. Engl. J. Med..

[44] Liu Z., Yu P., Dong L., Wang W., Duan S., Wang B., Gong X., Ye L., Wang H., Tian J. (2021). Discovery
of the next-generation pan-TRK kinase inhibitors for the treatment
of cancer. J. Med. Chem..

[45] Sharma V., Gupta M. (2022). Designing of kinase hinge binders:
A medicinal chemistry perspective. Chem. Biol.
Drug Des..

[46] Mammoliti O. (2024). Discovery of GLPG3667,
a selective ATP competitive tyrosine kinase
2 inhibitor for the treatment of autoimmune disease. J. Med. Chem..

[47] Ertl P., Schuffenhauer A. (2009). Estimation of synthetic accessibility score of drug-like
molecules based on molecular complexity and fragment contributions. J. Cheminf..

[48] Chen Z., Zhang L., Zhang P., Guo H., Zhang R., Li L., Li X. (2024). Prediction of Cytochrome
P450 Inhibition Using a Deep
Learning Approach and Substructure Pattern Recognition. J. Chem. Inf. Model..

[49] Bickerton G. R., Paolini G. V., Besnard J., Muresan S., Hopkins A. L. (2012). Quantifying
the chemical beauty of drugs. Nat. Chem..

[50] Akiyama H. (2000). Inflammation and Alzheimer’s disease. Neurobiol. Aging.

[51] Kinney J. W., Bemiller S. M., Murtishaw A. S., Leisgang A. M., Salazar A. M., Lamb B. T. (2018). Inflammation as
a central mechanism in Alzheimer’s
disease. Alzheimer’s Dementia.

[52] Leng F., Edison P. (2021). Neuroinflammation and
microglial activation in Alzheimer
disease: where do we go from here?. Nat. Rev.
Neurol..

[53] Tansey M. G., Wallings R. L., Houser M. C., Herrick M. K., Keating C. E., Joers V. (2022). Inflammation and immune
dysfunction in Parkinson disease. Nat. Rev.
Immunol..

[54] Vande
Walle L., Lamkanfi M. (2024). Drugging the NLRP3 inflammasome:
from signalling mechanisms to therapeutic targets. Nat. Rev. Drug Discovery.

[55] Swanson K. V., Deng M., Ting J. P.-Y. (2019). The NLRP3 inflammasome:
molecular
activation and regulation to therapeutics. Nat.
Rev. Immunol..

[56] Yao J., Wang Z., Song W., Zhang Y. (2023). Targeting NLRP3 inflammasome
for neurodegenerative disorders. Mol. Psychiatry.

[57] Li N., Zhang R., Tang M., Zhao M., Jiang X., Cai X., Ye N., Su K., Peng J., Zhang X., Wu W., Ye H. (2023). Recent progress
and prospects of small molecules for
NLRP3 inflammasome inhibition. J. Med. Chem..

[58] Nizami S., Millar V., Arunasalam K., Zarganes-Tzitzikas T., Brough D., Tresadern G., Brennan P. E., Davis J. B., Ebner D., Di Daniel E. (2021). A phenotypic
high-content, high-throughput
screen identifies inhibitors of NLRP3 inflammasome activation. Sci. Rep..

[59] Dalke A., Hert J., Kramer C. (2018). Mmpdb: An
open-source matched molecular
pair platform for large multiproperty data sets. J. Chem. Inf. Model..

[60] Coll R. C. (2015). A small-molecule inhibitor
of the NLRP3 inflammasome for the treatment
of inflammatory diseases. Nat. Med..

[61] Ritchie T. J., Macdonald S. J. F. (2016). Heterocyclic replacements for benzene: Maximising ADME
benefits by considering individual ring isomers. Eur. J. Med. Chem..

[62] Dossetter A. G., Douglas A., O’Donnell C. (2012). A matched
molecular pair analysis
of in vitro human microsomal metabolic stability measurements for
heterocyclic replacements of di-substituted benzene containing compounds
– identification of those isosteres more likely to have beneficial
effects. MedChemComm.

[63] Armstrong M. S., Morris G. M., Finn P. W., Sharma R., Moretti L., Cooper R. I., Richards W. G. (2010). ElectroShape: fast molecular similarity
calculations incorporating shape, chirality and electrostatics. J. Comput. Aided Mol. Des..

[64] Wójcikowski M., Zielenkiewicz P., Siedlecki P. (2015). Open Drug
Discovery Toolkit (ODDT):
a new open-source player in the drug discovery field. J. Cheminf..

[65] Patel H. M., Sing B., Bhardwaj V., Palkar M., Shaikh M. S., Rane R., Alwan W. S., Gadad A. K., Noolvi M. N., Karpoormath R. (2015). Design, synthesis
and evaluation of small molecule
imidazo­[2,1-b]­[1,3,4]­thiadiazoles as inhibitors of transforming growth
factor-β type-I receptor kinase (ALK5). Eur. J. Med. Chem..

[66] Ramprasad J., Nayak N., Dalimba U., Yogeeswari P., Sriram D., Peethambar S. K., Achur R., Kumar H. S. S. (2015). Synthesis
and biological evaluation of new imidazo­[2,1-b]­[1,3,4]­thiadiazole-benzimidazole
derivatives. Eur. J. Med. Chem..

[67] Ramprasad J., Nayak N., Dalimba U., Yogeeswari P., Sriram D. (2016). Ionic liquid-promoted one-pot synthesis of thiazole–imidazo­[2,1-b]­[1,3,4]­thiadiazole
hybrids and their antitubercular activity. Medchemcomm.

[68] Meanwell N. A., Sistla R., Lolli M., Meanwell N. (2021). Chapter Two - A survey
of applications of tetrahydropyrrolo-3,4-azoles and tetrahydropyrrolo-2,3-azoles
in medicinal chemistry. Adv. Heterocycl. Chem..

[69] Hoffmann-La Roche AG, F. Bicyclic ketone compounds and methods of use thereof. European Patent EP 3652178 A1, 2020.

[70] Hoffmann-La Roche AG, F. Bicyclic lactams as receptor-interacting protein-1 (RIP1) kinase inhibitors for treating e.g. inflammatory diseases. European Patent EP 3760625 A1, 2021.

[71] Seneci P., Nicola M., Inglesi M., Vanotti E., Resnati G. (1999). Synthesis
of mono- and disubstituted 1H-imidazo [1,2-b] pyrazoles. Synth. Commun..

[72] Sagar R., Mishra V. K., Tiwari G., Khanna A., Tyagi R. (2024). Efficient
synthesis of chirally enriched 1H-imidazo­[1,2-b]­pyrazole- and 4H-imidazo­[1,2-b]­[1,2,4]­triazole-based
bioactive glycohybrids. Synthesis.

[73] Wang J.-L., Wu G.-Y., Luo J.-N., Liu J.-L., Zhuo C.-X. (2024). Catalytic
intermolecular deoxygenative coupling of carbonyl compounds with alkynes
by a Cp*Mo­(II)-catalyst. J. Am. Chem. Soc..

[74] Lapsien M., Bonus M., Gahan L., Raguin A., Gohlke H. (2025). PyPE_RESP:
A tool to facilitate and standardize derivation of RESP charges. J. Chem. Inf. Model..

[75] Coley C. W., Rogers L., Green W. H., Jensen K. F. (2018). SCScore: synthetic
complexity learned from a reaction corpus. J.
Chem. Inf. Model..

[76] Thakkar A., Chadimová V., Bjerrum E. J., Engkvist O., Reymond J.-L. (2021). Retrosynthetic
accessibility score (RAscore) – rapid machine learned synthesizability
classification from AI driven retrosynthetic planning. Chem. Sci..

