# Supplementary material for: The Heterocycle Isostere Explorer: A Computational Tool for the Discovery of Novel Aromatic Heterocyclic Isosteres
Source: J Med Chem. 2026 Feb 11;69(4):4408–23. doi: 10.1021/acs.jmedchem.5c03118 (PMC12951576; doi:10.1021/acs.jmedchem.5c03118)
Supplement: Supplementary file 1 [file jm5c03118_si_001.pdf]

Supporting Information

The Heterocycle Isostere Explorer: A Computational Tool for the Discovery of Novel Aromatic Heterocyclic Isosteres

Matthew T. O. Holland[†, ‡], Víctor Sebastián-Pérez[¶,⊥], Anthony R. Bradley[§,||], Fernanda Duarte[‡], Paul E. Brennan[*,†]

[†]Centre for Medicines Discovery, University of Oxford, NDM Research Building, Old Road Campus, Oxford, OX3 7FZ (UK)

[‡]Chemistry Research Laboratory, University of Oxford, Mansfield Road, Oxford, OX1 3TA (UK)

[¶]Exscientia Plc., The Schrödinger Building, Oxford Science Park, Oxford, OX4 4GE (UK)

[§]Department of Chemistry, University of Liverpool, Crown Street, Liverpool, L69 7ZD (UK)

[||]Department of Computer Science, University of Liverpool, Brownlow Hill, Liverpool, L69 7ZX (UK)

[⊥]*Present address:* SandboxAQ, 780 High Street, Palo Alto, CA 94301 (USA)

*Correspondence: `paul.brennan@cmd.ox.ac.uk`

Contents

S-1 Filtering the Dataset to Generate MoBiVic S2
S-2 Properties of MoBiVic and VEHICLe S3
S-3 Benchmarking of Geometry Optimisation and Partial Charge Calculations S5
S-4 Kabsch-Umeyama Algorithm for Heterocyclic Alignment S15
S-5 Determining the Bin Boundaries for Hash Generation S17
S-6 Runtime Scaling S19
S-7 NLRP3 Correlations S21
S-8 3,5-Disubstituted pyrazolo[1,5-a]pyrimidine SwissBioisostere Results S23
S-9 VEHICLe Sample for Geometry RMSD Benchmarking S25
S-10 Molecular Context Benchmarking S26

S-1 Filtering the Dataset to Generate MoBiVic

After mono- and bi-functionalising the VEHICLe dataset with the substituent set described in the main text, the resulting complete dataset of heterocycles was filtered to remove molecules that were likely to be unstable. The filters used to identify and remove these molecules are given in the main body of the text, and are listed in Figure S1 below alongside examples of molecules removed for each filter. The SMARTS expressions used to filter the dataset are given for each rule. The full dataset and the filtered dataset (MoBiVic) are available on GitHub, along with the script used to generate and filter the datasets.

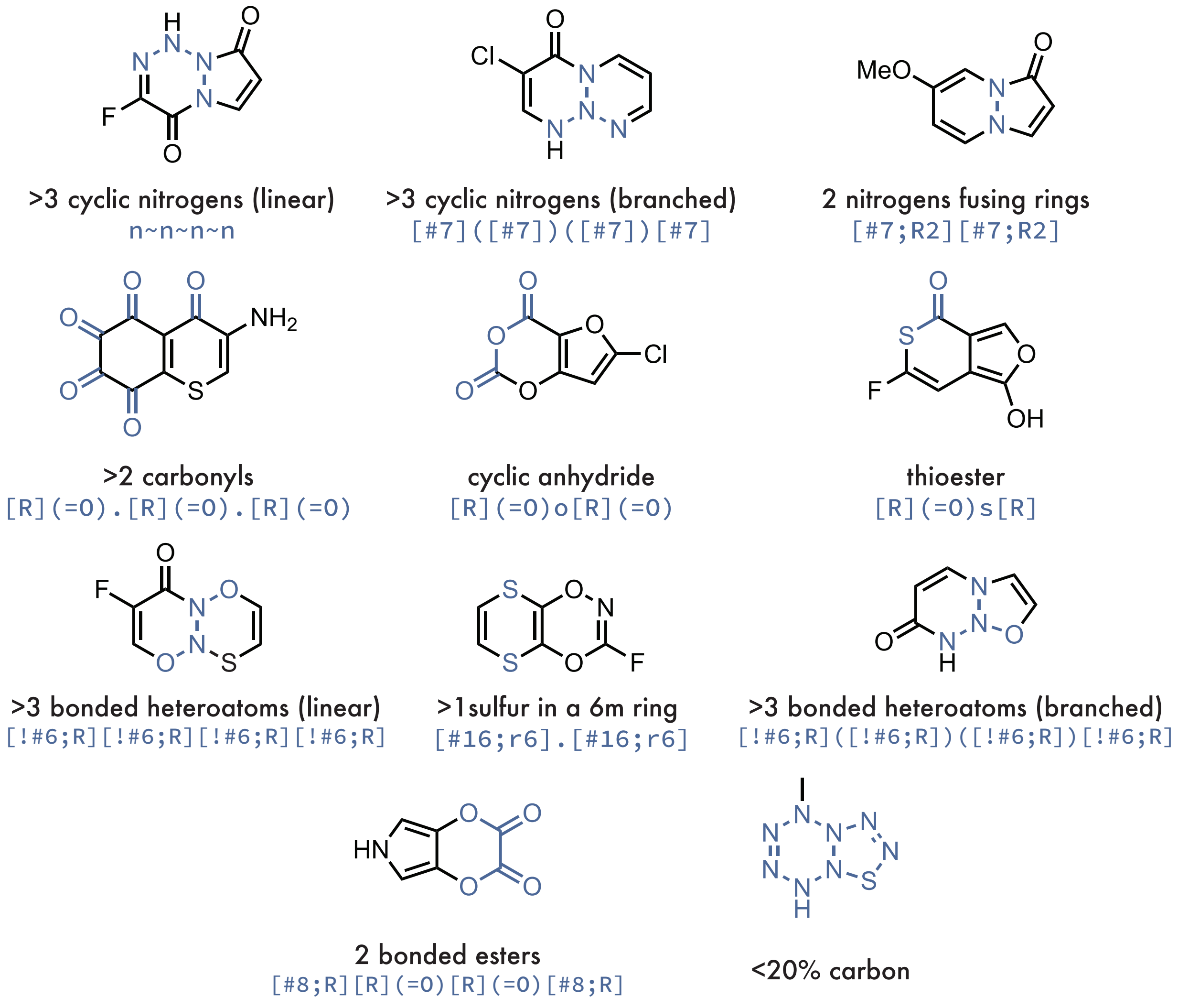

Figure S1: The filters used to remove 'potentially explosive or bonkers' molecules from the dataset. Examples of molecules removed from the dataset for each filter are given, with the pattern leading to their removal highlighted in blue. The SMARTS expression for each filter is given in blue below the molecule.

S-2 Properties of MoBiVic and VEHICLe

S-2.1 Physicochemical Properties of MoBiVic and VEHICLe

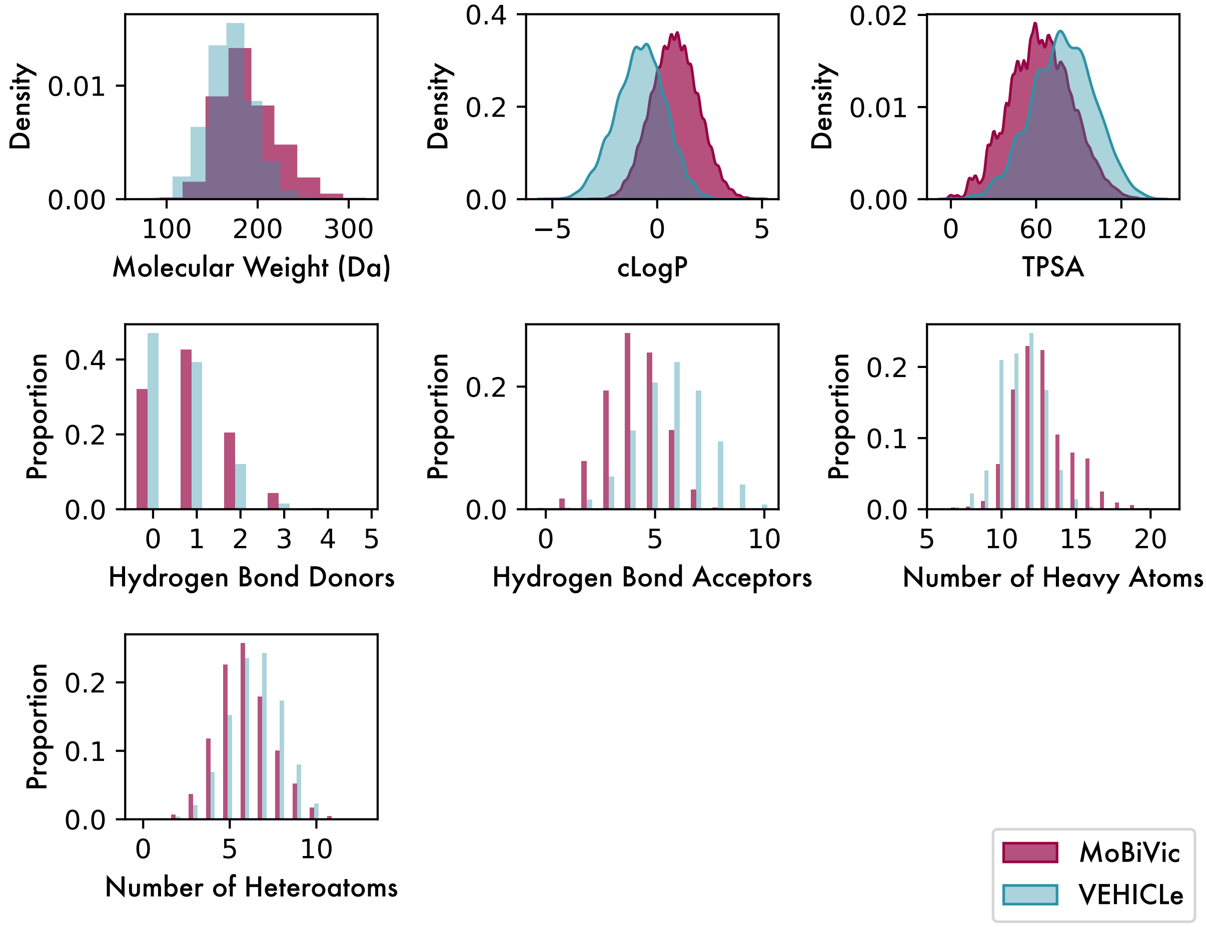

Figure S2: A comparison of the medicinally-relevant physicochemical properties of MoBiVic and VEHICLe.

S-2.2 SYBA Scores of MoBiVic Heterocycles

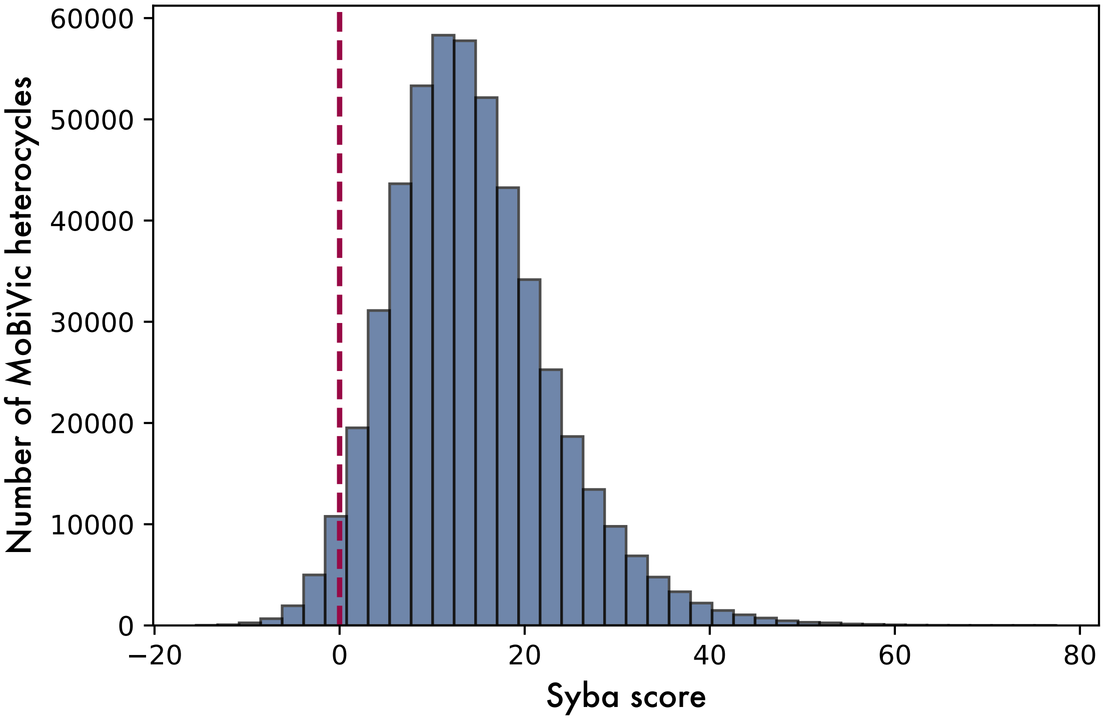

Figure S3: The distribution of SYBA scores across the MoBiVic library, as calculated using the Python SybaClassifier with default scoring. A SYBA score > 0 (indicated by the dotted red line) indicates that the local atomic environments in the heterocycle are statistically common in previously synthesised molecules. Over 97% of the MoBiVic heterocycles had SYBA scores > 0, suggesting that the heterocycles are largely composed of fragment patterns that frequently appear in already synthesised molecules. Furthermore, a negative SYBA score does not imply that a molecule cannot be synthesised, only that the pattern of its atoms and bonding is not common in already synthesised molecules.

S-3 Benchmarking of Geometry Optimisation and Partial Charge Calculations

In order to maximise user customisability and the flexibility of the software, we opted not to pre-calculate any partial charges or geometries for the MoBiVic molecules. Instead, all necessary molecular properties were calculated dynamically at runtime, allowing users to apply their own methods or parameters to the database to meet the requirements of their respective projects. This also allowed us to minimise the number of external packages required by the software. The charges and geometries of the user-specified query molecules have to be calculated using the same method as those of the MoBiVic molecules if a comparison is to be meaningful, thus using high-level electronic structure methods would have necessitated bundling or requiring third-party quantum chemistry packages, many of which are commercial or have restrictive licenses.

S-3.1 Geometry Optimisation

We wished for the default baseline methods of geometry optimisation and partial charge calculation to be computationally efficient and ideally distributed as part of an open-source Python package. RDKit is used within the software for handling virtual instances of molecules, and comes with a built-in geometry optimisation algorithm that avoids expensive quantum mechanical calculations by using an experimental torsion-knowledge distance geometry (ETKDG) algorithm.[1] This algorithm generates realistic geometries for small molecules by initially creating a random estimate of the bond lengths in the molecule using constraints derived from crystallographic data, before refining these using experimentally-derived knowledge of bond lengths, bond angles, and dihedral angles. Although the RDKit developers claim that the geometries generated from this algorithm are good enough for most computational purposes without further refinement, it is often standard practice to further optimise ETKDG-generated geometries with a molecular force field.

To benchmark the validity of the ETKDG optimisations, and to assess both the necessity and feasibility of a further MMFF optimisation on the heterocycles, we took a random sample of 250 MoBiVic heterocycles (the 'testing set'; the RegIDs of these are given in Table S11) and optimised their geometries using a variety of methods representing a range of levels of theory, and calculated the RMSDs of these relative to the ETKDG geometries (see Figure S4). We also compared the average time taken per molecule for each of these methods (see Table S1). Each molecule had an initial geometry generated by ETKDG, as implemented in RDKit, and these geometries were further refined using the MMFF94 molecular force field, the semi-empirical xTB approach developed by Grimme *et al.*, and using DFT (PBE0 functional with D3BJ dispersion correction) at a medium (def2-TZVP basis set) level of theory.[2]

It is clear from the data in Table S1 that the DFT-based optimisation is significantly slower than the ETKDG and MMFF94 parametrised models, taking over an hour per molecule. The xTB approach is substantially faster than any of the ab-initio DFT methods, but is orders of magnitude slower than the MMFF94 and ETKDG methods.

Table S1: The average time taken (per molecule) to optimise the molecular geometries across the various methods used in benchmarking the geometry optimisations.

Method	Average time per molecule
ETKDG	3.7 ms
MMFF94	420 µs
xTB	246 ms
D3BJ-PBE0/def2-TZVP	1 hour 39 minutes

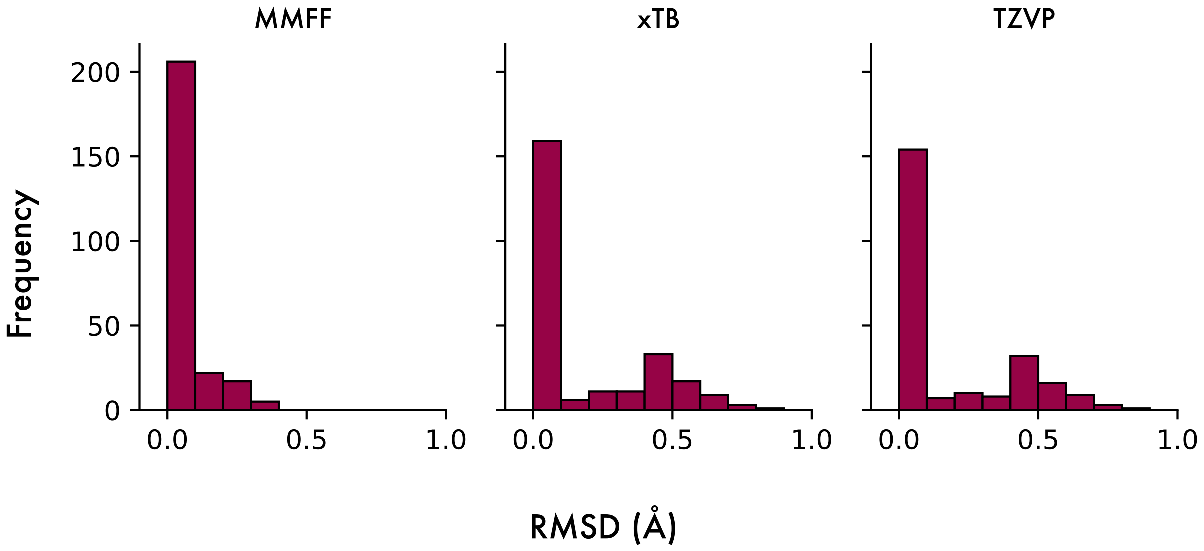

Figure S4: The RMSDs for the molecular geometries generated by each higher-level optimisation method relative to the geometry generated by the ETKDG algorithm implemented in RDKit. The D3BJ-PBE0 functional was used for the DFT calculation, and the label on this plot refers to the basis set used.

The results in Figure S4 show that the geometries after MMFF optimisation are most similar to those generated by the ETKDG method, with 82% of the RMSDs < 0.1 Å. The range of RMSDs is greater when the xTB and DFT methods are compared; these methods having RMSDs between 0.0 – 1.0 Å. Pleasingly all RMSDs are below 1.0 Å, and the majority of the RMSDs (64% for xTB and 62% for DFT) for both higher methods are < 0.1 Å. Riniker *et al.* use a 1.0 Å cut-off as a threshold for deciding whether conformers of the same molecule are different, and it is common in molecular docking to use 2.0 Å as an upper bound for deciding if a crystal structure has been successfully reproduced.[1,3] Therefore having the majority of molecules < 0.1 Å across the three methods is sufficient to conclude that the higher-level geometry optimisations offered by xTB and DFT are unnecessary in this context. Furthermore, the significant time penalty that these methods incur would render HCIE slow and inconvenient. Although the xTB method is substantially quicker, it is still nearly 100 times slower than a simple ETKDG optimisation alone, and its results are not significantly different to justify this time penalty. Therefore ETKDG initial geometries with a subsequent MMFF optimisation was selected as the default baseline geometry optimisation method.

S-3.2 Conformers

The MoBiVic heterocycles have low levels of conformational flexibility available to them due to high sp^2 proportions, with an average of 0.3 rotatable bonds per molecule. As a result of this, only one conformer is optimised and aligned for each heterocycle in the interests of computational efficiency, as other conformers are likely to be geometrically very similar. To confirm that this approach is appropriate, conformers were generated for the sample of 250 random MoBiVic heterocycles outlined above, and their geometries optimised and compared.

For each heterocycle in the sample, 25 conformers were generated using the `EmbedMultipleConfs` function as part of RDKit. Each conformer was optimised first using the ETKDG and then the MMFF algorithms, as above. For each heterocycle, the 25 conformers were compared to one another by calculating their pairwise heavy atom RMSDs. These RMSD values were then combined across all 250 heterocycles, and are displayed in Figure S5 below.

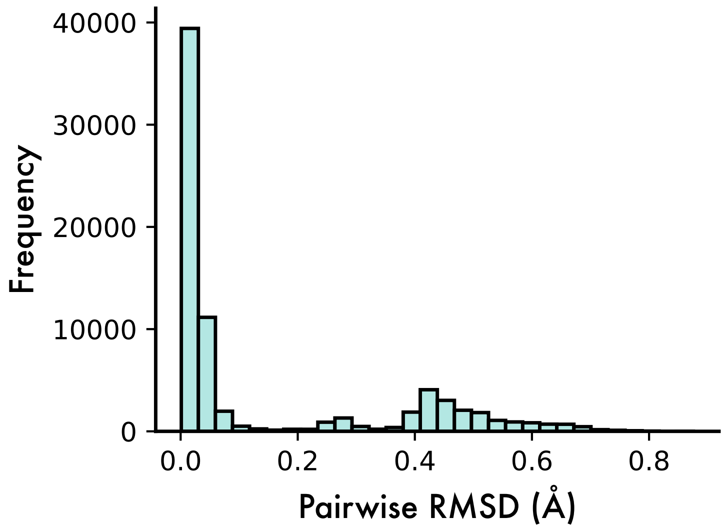

Figure S5: The pairwise heavy atom RMSDs between the 25 generated conformers for each of the 250 heterocycles in the random sample of MoBiVic.

It is clear from Figure S5 that all the conformers are geometrically similar to one another, with a mean RMSD across the whole sample of 0.15 Å. That 71% of the pairwise similarities have RMSDs $<$ 0.25 Å, 92% below 0.5 Å, and 99% are below 0.75 Å suggests that the geometric variation between conformers is small enough that considering only one low-energy conformation is an acceptable compromise for computational speed in this instance. Although there is no universally agreed cut-off for conformer similarity, Riniker *et al.* have previously used 1.0 Å as a cut-off, and thus having the overwhelming majority of conformers $<$ 0.75 Å is in keeping with this precedent.[1]

Examining Figure S5 reveals that there is a small number of conformer pairs with RMSDs between 0.4–0.6 Å. These correspond to heterocycles carrying *N*-trifluoroethyl substituents, and these increased RMSDs can be explained by the added degrees of conformational freedom conferred by

this more flexible substituent. Inspection of the conformer structures for these heterocycles revealed that the larger RMSD values were between conformers where the trifluoroethyl group took different orientations. The geometries of the rings were highly similar in all cases. Although only comparing a single conformer of these N-trifluoroethyl heterocycles when HCIE searching does lead to a more restricted sampling of the conformational space, the minor effect that this group has on the geometry of the rings in these cases, and the increased computational cost that involving multiple conformers would incur supports the compromise of using a single, representative low-energy conformer for all heterocycles in searching.

S-3.3 Partial Charges

Atomic partial charges are used within HCIE as a surrogate for molecular ESP. As with geometry optimisation, no partial charge information was exported with the MoBiVic library to ensure user customisability and flexibility, thus partial charges must be calculated dynamically at runtime. With a library of over 500 000 molecules, computationally expensive single point calculations to extract charge information would likely introduce large delays in the searching. Therefore the chosen partial charge method must balance efficiency with accuracy.

Within RDKit is implemented a simple and efficient method for approximating partial charges using an iterative method of orbital electronegativity equalisation described by Gasteiger and Marsili in 1980.[4] This involves iteratively distributing charge based on the bond order and electronegativity difference between atoms in bonds, until the partial charges are stable across the molecule. It does not involve electronic structure calculations and is geometry independent and so is very quick, taking on average 11 µs for the molecules in the testing set (see Table S2). As with the results for the geometry benchmarking, the xTB and DFT single points were substantially slower. These charges were also compared to those calculated using the Restrained Electrostatic Potential (RESP) method and the AM1-BCC partial charge method, which are popular in medicinal chemistry.[5–8] It is clear from Table S2 that although the RESP and AM1-BCC charges are faster than the ab initio DFT calculations, they are still orders of magnitude slower, on average, than the Gasteiger method.

Table S2: The average time taken per molecule to evaluate a single-point energy for the subset of 250 molecules randomly sampled from MoBiVic. The geometries used were those optimised by ETKDG followed by MMFF in RDKit.

Method	Average time per molecule
ETKDG	11 µs
xTB	341 ms
D3BJ-PBE0/def2-TZVP	6 minutes 56 s
RESP	6.41 s
AM1-BCC	1.46 s

To probe the suitability of the Gasteiger charges, partial charges were calculated for the same 250

MoBiVic heterocycles using the electronic structure methods in Table S2, and these compared to the charges derived by Gasteiger's method (see Figure S6). Inspection of the plots in Figure S6 show there to be a clear positive correlation between the charges calculated by Gasteiger's method, and those by xTB and TZVP, with Pearson correlation coefficients of 0.89 and 0.83 respectively. Correlation matrices between the charge methods, calculated using both the Pearson correlation coefficient and Spearman's rank correlation coefficient, are shown in Figure S7. This shows that the level of correlation across the charge methods is good, with all methods displaying correlations > 0.8 that are consistent across the two metrics.

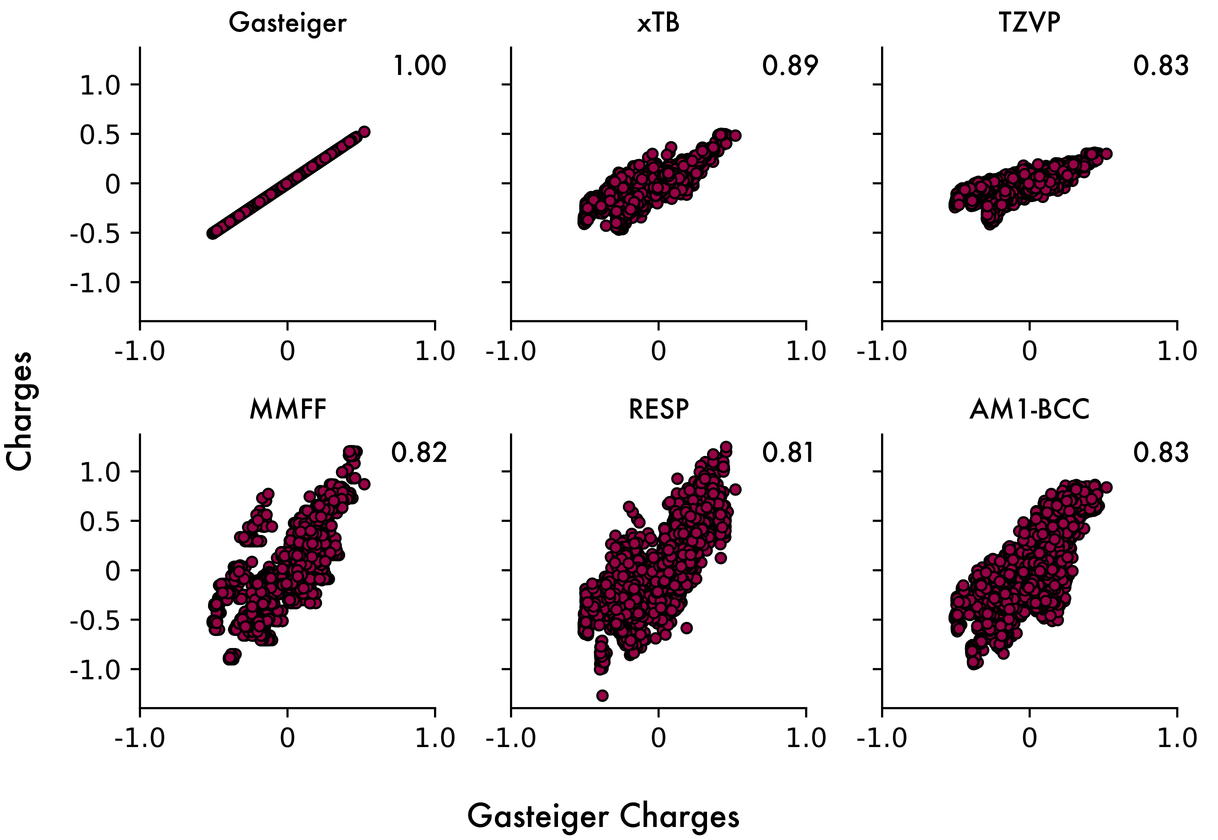

Figure S6: A comparison of the partial charges for the random subset of MoBiVic calculated using various electronic structure methods spanning low and high levels of theory to those calculated using Gasteiger's method. The Pearson correlation coefficients for the charges compared to the Gasteiger charges are given for each method in the upper right corner.

The charges calculated using the Merck Molecular Force Field (MMFF; as implemented in RDKit), RESP, and AM1-BCC do display a positive correlation with the Gasteiger charges, but the range of charges calculated using these three methods is far broader than those calculated using Gasteiger, xTB, and DFT methods. It is clear from Figure S8 that charges close to ± 1 are calculated by these methods, which are outside of the range typically expected for atomic partial charges in neutral organic molecules. Table S3 shows that the proportion of the atoms in the sample calculated to have these large charges is significantly higher for the MMFF, RESP, and AM1-BCC methods than

for the other three methods. This suggests that these partial charge models calculate a greater prevalence of large atomic charges, and as such may not accurately reflect the underlying electronic structure for the MoBiVic heterocycles.

Table S3: The number of atoms in the random subset with absolute partial charges greater than 0.5 or 0.7, and the proportion of the atoms in the random subset that this represents, for each partial charge method.

	$\|q\| > 0.5$		$\|q\| > 0.7$	
Method	Count	Proportion	Count	Proportion
Gasteiger	26	1%	0	0%
xTB	0	0%	0	0%
TZVP	0	0%	0	0%
MMFF	1010	23%	259	6%
RESP	814	19%	202	5%
AM1-BCC	888	20%	220	5%

To ensure that the deviations from perfect correlation with the Gasteiger charges were not due to systematic biases related to atomic properties, the charges were plotted by element (Figure S8a) and by hybridisation state (Figure S8b). These analyses revealed no consistent element-specific or hybridisation-dependent trends that would indicate a systematic deviation between Gasteiger and quantum-chemically-derived charges.

As atomic partial charges are not observable quantities, there is no 'ground truth' to compare these charges to. However, the significant efficiency advantages that the Gasteiger charges provide, when taken in the context of the strong correlations with higher-level charges illustrated in Figure S8, justify their use as the baseline charge method in HCIE. Should users require higher-level quantum chemical calculations for their applications, it is straightforward to incorporate these into the codebase.

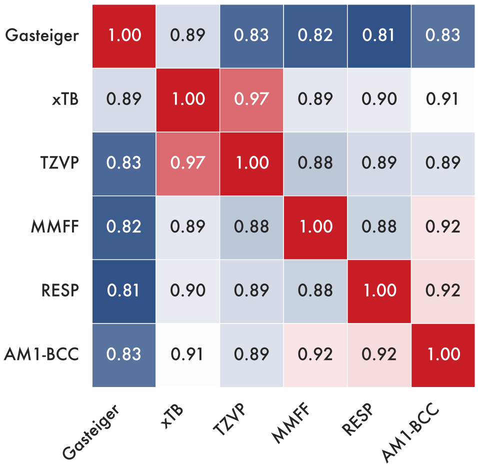

(a) Pearson correlation coefficients.

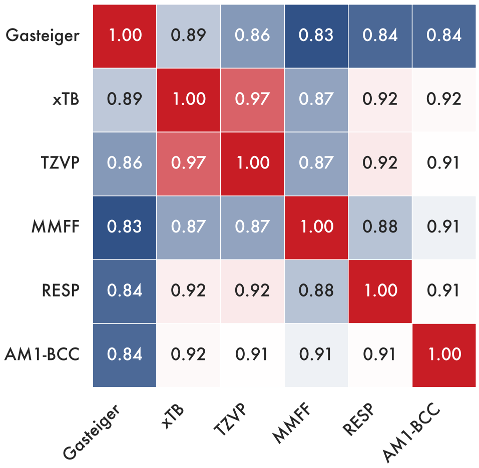

(b) Spearman's rank correlation coefficients.

Figure S7: Comparison of charge-model correlations.

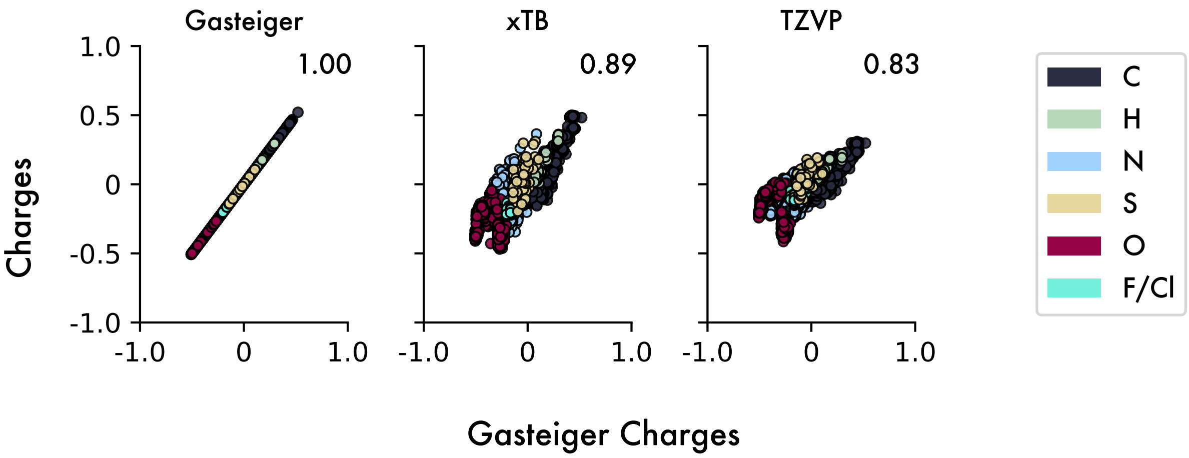

(a) A comparison of the partial charges for the random subset of MoBiVic, coloured by element. The Pearson correlation coefficients for the charges compared to those calculated by Gasteiger's method are given in the upper right corner.

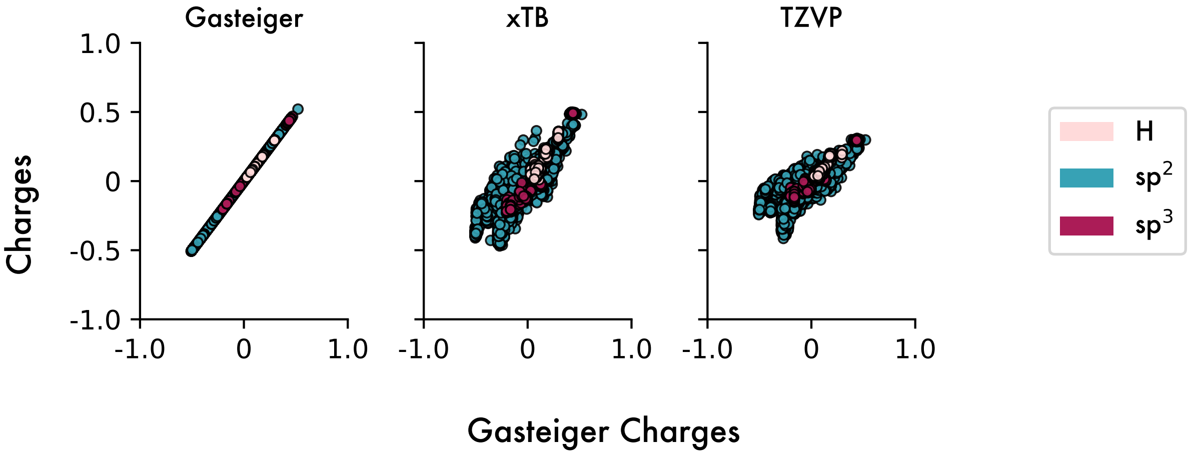

(b) The same partial charges, now coloured by atomic hybridisation state.

Figure S8: Partial charge comparisons for a random subset of MoBiVic.

S-3.3.1 Benchmarking against RESP and AM1-BCC Charges

To validate the sensitivity of the HCIE methodology to the choice of atomic partial charges, we performed a controlled comparison of HCIE's default Gasteiger charges to those calculated using RESP and AM1-BCC models. A random subset of 50 MoBiVic heterocycles was sampled linearly across the RegIDs, thereby ensuring that the sample was representative of the structures and substitutions found in the library. RESP and AM1-BCC charges were calculated separately (see above) for each molecule in the sample, and a database assembled with this information. One molecule (S235960; SMILES: `Cn2cc[nH]c1c([R])oncn12`) from this sample was selected at random as the query, and the sample of 50 used as the searchable database. For each charge model, a one-vector HCIE search was performed using identical geometric alignment and shape similarity settings, differing only in the source of atomic partial charges used to compute electrostatic similarity. Table S4 shows the overlap of the top 20 molecules of each charge method with those of the Gasteiger search. For illustrative purposes the RegIDs of the top 20 molecules returned from the Gasteiger search vs the AM1-BCC search is shown in Table S5.

Table S4: Comparison of HCIE one-vector search rankings obtained using different partial charge models, using the Gasteiger-charge search as the reference.

Charge model	Top-20 overlap with Gasteiger	Spearman's ρ (vs Gasteiger)
RESP	15	0.89
AM1-BCC	15	0.88

The substantial agreement observed between rankings obtained with RESP, AM1-BCC, and Gasteiger charges indicates that HCIE's electrostatic similarity scoring is primarily sensitive to relative charge distributions rather than the absolute values produced by a particular charge model. Despite the fundamentally different physical approximations underlying RESP, AM1-BCC, and Gasteiger charges, the high rank correlations (Spearman's $\rho \approx 0.9$) and the recovery of 15 out of the top 20 Gasteiger-ranked candidates demonstrate that candidate prioritisation is largely preserved. Given that Gasteiger charges can be generated rapidly at runtime and scale efficiently to the full MoBiVic database, these results support their use as a pragmatic and computationally efficient choice for large-scale HCIE searches. Importantly, HCIE's implementation permits the substitution of alternative charge models, enabling users to balance computational cost and electrostatic fidelity according to their project's requirements.

S-3.3.2 Methodology

To calculate RESP charges, molecular geometries were taken from structures optimised by HCIE, and single-point electrostatic potentials were computed at the HF/6-31G* level using Gaussian 16. Atomic partial charges were then obtained by fitting to the electrostatic potential using the RESP method as implemented in antechamber (AmberTools), with standard hyperbolic restraints.[9,10]

AM1-BCC charges were computed for the same geometries using the antechamber module of Am-

berTools, employing semiempirical AM1 Mulliken charges followed by the application of predefined bond charge corrections, according to the original AM1-BCC parametrisation.

Table S5: Top 20 HCIE-ranked MoBiVic heterocycles for query RegID S235960, comparing Gasteiger and AM1-BCC partial charges, using the random sample of 50 heterocycles as the searchable library. Entries shown are RegID and total score (shape + ESP; range 0–2).Shaded entries are present in both top-20 lists, the Spearman's ρ correlations are given in Table S4.

	Gasteiger		AM1-BCC	
Rank	RegID	Total score	RegID	Total score
1	S235960	2.00	S235960	2.00
2	S373155	1.35	S373155	1.48
3	S810969	1.10	S468155	1.29
4	S611375	0.97	S680701	1.27
5	S680701	0.92	S611375	1.11
6	S294066	0.87	S594883	0.99
7	S174692	0.86	S475655	0.95
8	S171260	0.85	S294066	0.94
9	S570664	0.79	S174692	0.94
10	S731050	0.79	S171260	0.94
11	S223214	0.79	S48556	0.94
12	S475655	0.79	S136268	0.90
13	S770034	0.79	S770034	0.88
14	S791564	0.78	S223214	0.85
15	S735514	0.77	S570664	0.84
16	S642435	0.77	S731050	0.84
17	S494281	0.77	S735514	0.83
18	S557844	0.76	S494281	0.83
19	S583522	0.76	S2905	0.82
20	S241081	0.75	S241081	0.82

S-4 Kabsch-Umeyama Algorithm for Heterocyclic Alignment

For each exit-vector in the probe molecule the non-H atom (herein referred to as the base atom) is translated to the origin. The base atom of the user-specified exit-vector in the query atom is also translated to the origin. As only two vectors are needed to define the plane of an aromatic ring, the matrix of coordinates required for calculating the rotation can be reduced to just three of the ring atoms (the base atom, and its neighbouring two ring atoms). The coordinates of these (after translation) are used to form the matrices needed for alignment, as illustrated in S9b. The **P** matrix is formed of the coordinates of the requisite atoms from the probe molecule, and the **Q** matrix from those of the query molecule.

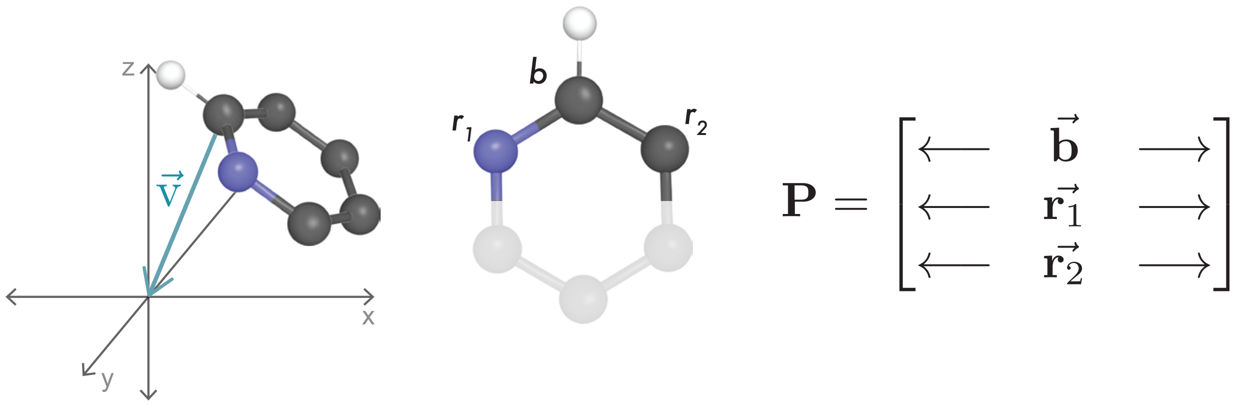

(a) Centering the base atom of the vector pair at the origin. This acts as the centre of rotation. The vector ($\vec{v}$) defines the translation of the base atom to the origin.

(b) The construction of the **P** matrix for the 2-pyridine exit-vector. **b** defines the base atom of the exit-vector, and $\mathbf{r}_1$ and $\mathbf{r}_2$ are the ring atoms that define the plane of the ring. Their coordinates make up the rows of the **P** matrix.

Figure S9: The components of the Kabsh-Umeyama algorithm for aligning each probe onto the query heterocycle.

The optimal rotation matrix that rotates the points defined in **P** onto the points defined in **Q** such that the RMSD between them is minimised is then found by first determining the co-variance matrix

$$\mathbf{H} = \mathbf{P}^{\mathrm{T}}\mathbf{Q}$$

which captures the degree of alignment and spatial correlation between the points in each set. This is then deconstructed into its component matrices using a singular value decomposition

$$\mathbf{H} = \mathbf{U}\mathbf{\Sigma}\mathbf{V}^{\mathrm{T}}$$

where $\mathbf{U}$ and $\mathbf{V}^{\mathrm{T}}$ are orthogonal matrices representing rotations, and $\mathbf{\Sigma}$ is a diagonal matrix

representing a scaling. Finally, the matrix that represents the optimum rotation is found

$$\mathbf{R} = \mathbf{V} \begin{pmatrix} 1 & 0 & 0 \\ 0 & d & 0 \\ 0 & 0 & 1 \end{pmatrix} \mathbf{U}^{\mathrm{T}} \quad \text{where } d = \det(\mathbf{V}\mathbf{U}^{\mathrm{T}}) = \begin{cases} +1 & \text{if rotation} \\ -1 & \text{if reflection} \end{cases}$$

The single value decomposition can, depending on the precise nature of $\mathbf{P}$ and $\mathbf{Q}$, produce a matrix that corresponds to a reflection rather than a rotation. The inclusion of d in the identity matrix multiplication corrects for this occurrence.

This rotation matrix $\mathbf{R}$ is then applied to the full matrix of the probe's coordinates, to generate an alignment to the query $\mathbf{P}_{\text{aligned}}$, which is then scored as described above. $\mathbf{P}_{\text{aligned}}$ is then multiplied by a matrix corresponding to a 180° rotation about the axis defined by the probe exit-vector, and re-scored. This 180° rotation matrix is defined using the Euler-Rodrigues formula.

S-5 Determining the Bin Boundaries for Hash Generation

To determine the positions of the boundaries for the two-vector hash bins, the distribution of distances (d) and angles (α_v) for heterocycles in the VEHICLe database was inspected, and boundaries drawn from these distributions. These are displayed in Figure S10. The distance and angle bins are listed in Table S6 and Table S7.

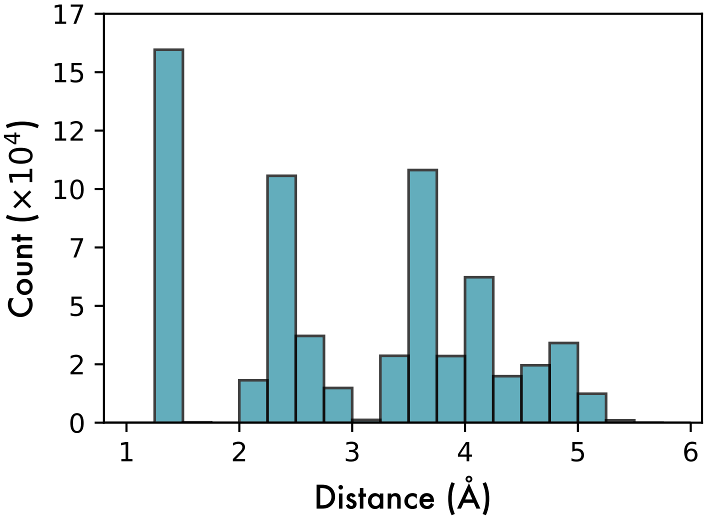

(a) A histogram of the distances between base atoms in the VEHICLe database.

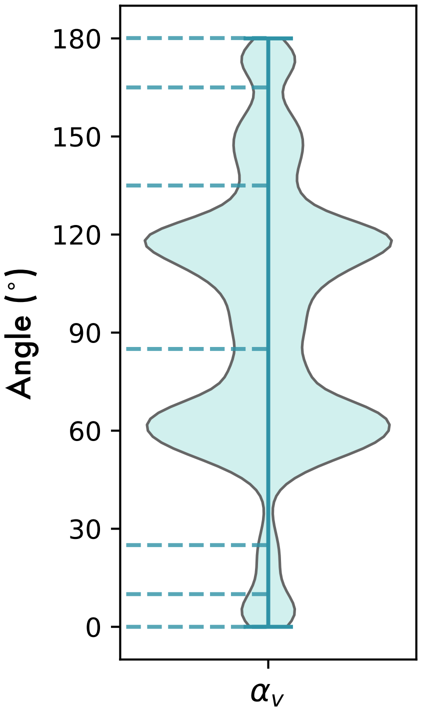

(b) The angles between exit-vector pairs in the VEHICLe database. The horizontal lines indicate the chosen bin boundaries.

Figure S10: The distributions of two-vector parameters for the expanded database of heterocycles.

Table S6: The distance bins and their hash code.

Bin (d in Å)	Hash
$0 \leq d \leq 2$	00000
$2.00 \leq d < 2.25$	00001
$2.25 \leq d < 2.50$	00010
$2.50 \leq d < 2.75$	00011
$2.75 \leq d < 3.00$	00100
$3.00 \leq d < 3.25$	00101
$3.25 \leq d < 3.50$	00110
$3.50 \leq d < 3.75$	00111
$3.75 \leq d < 4.00$	01000
$4.00 \leq d < 4.25$	01001
$4.25 \leq d < 4.50$	01010
$4.50 \leq d < 4.75$	01011
$4.75 \leq d < 5.00$	01100
$5.00 \leq d < 5.25$	01101
$5.25 \leq d < 5.50$	01110
$5.50 \leq d < 5.75$	01111
$5.75 \leq d < 6.00$	10000
$6.00 \leq d < \infty$	10001

Table S7: The angle bins and their hash code.

Angle	Hash
$0° \leq \alpha_{\mathrm{v}} < 10°$	000
$10° \leq \alpha_{\mathrm{v}} < 25°$	001
$25° \leq \alpha_{\mathrm{v}} < 85°$	010
$85° \leq \alpha_{\mathrm{v}} < 135°$	011
$135° \leq \alpha_{\mathrm{v}} < 165°$	100
$165° \leq \alpha_{\mathrm{v}} \leq 180°$	101

S-6 Runtime Scaling

To characterise the runtime of a HCIE search as a function of the size of the database, we benchmarked the search procedure on an Apple M1 Pro (8-core CPU, 16 GB RAM) using randomly selected subsets of 10 000, 100 000, and the full MoBiVic library (500 968 heterocycles). 2-Pyridine was selected as the query ligand, and for each subset size the complete search workflow was executed five times with identical parameters, and the wall-clock runtimes were recorded. The resulting mean runtimes and standard deviations are reported in Table S8. These data show that HCIE exhibits approximately linear scaling with respect to the number of heterocycles searched, which is consistent with the design of the alignment and scoring algorithm.

Table S8: The average runtimes and standard deviations, in seconds, for five repeat searches of 2-pyridine with a randomly selected library of 10 000 and 100 000 heterocycles, and the full MoBiVic (500 968 heterocycle) database.

Library size	Average time (s)
10 000	75 ± 2
100 000	133 ± 1
MoBiVic	677 ± 6

The complete code to reproduce this analysis is included below.

```
import time
import csv
from datetime import datetime
from hcie import DatabaseSearch

def timed_run(vs, database_size: int) -> float:
    t0 = time.perf_counter()
    elapsed = vs.search(database_size=database_size)
    t1 = time.perf_counter()
    return elapsed

def run_benchmark(smiles: str, name: str | None = None):
    sizes = [10_000, 100_000, 500_968]
    repeats = 5
    results = []

    print(f"running benchmark for {smiles}")

    csv_filename = f"runtime_benchmark_{(name or 'query').replace(' ', '_')}.csv"

    with open(csv_filename, "w", newline="") as f:
        writer = csv.writer(f)

        # Header row
        writer.writerow([
```

```
            "timestamp",
            "query_smiles",
            "database_size",
            "run_index",
            "elapsed_seconds",
        ])

        for n in sizes:
            times = []
            for run_idx in range(1, repeats + 1):
                print(f"  size={n}, run={run_idx}/{repeats}")
                vs = DatabaseSearch(smiles=smiles, name=f"benchmark_{n}")
                t = timed_run(vs, database_size=n)
                times.append(t)

                # Write one line to CSV as soon as it is generated
                writer.writerow([
                    datetime.now().isoformat(timespec="seconds"),
                    smiles,
                    n,
                    run_idx,
                    f"{t:.2f}",
                ])
                f.flush()  # ensure results are saved to disk

            mean_t = sum(times) / repeats
            sd_t = (sum((t - mean_t) ** 2 for t in times) / repeats) ** 0.5
            results.append((n, mean_t, sd_t))

    print("Summary (size, mean_s, sd_s):")
    print(results)
    return results

if __name__ == "__main__":
    run_benchmark(smiles='[R]c1ccccn1', name='2-pyridine')
```

S-7 NLRP3 Correlations

Figure S11 shows the correlations of the 5 NLRP3 inhibitor matched molecular series both before and after the weightings were optimised. The series labels for each row correspond to those in Figure 8.

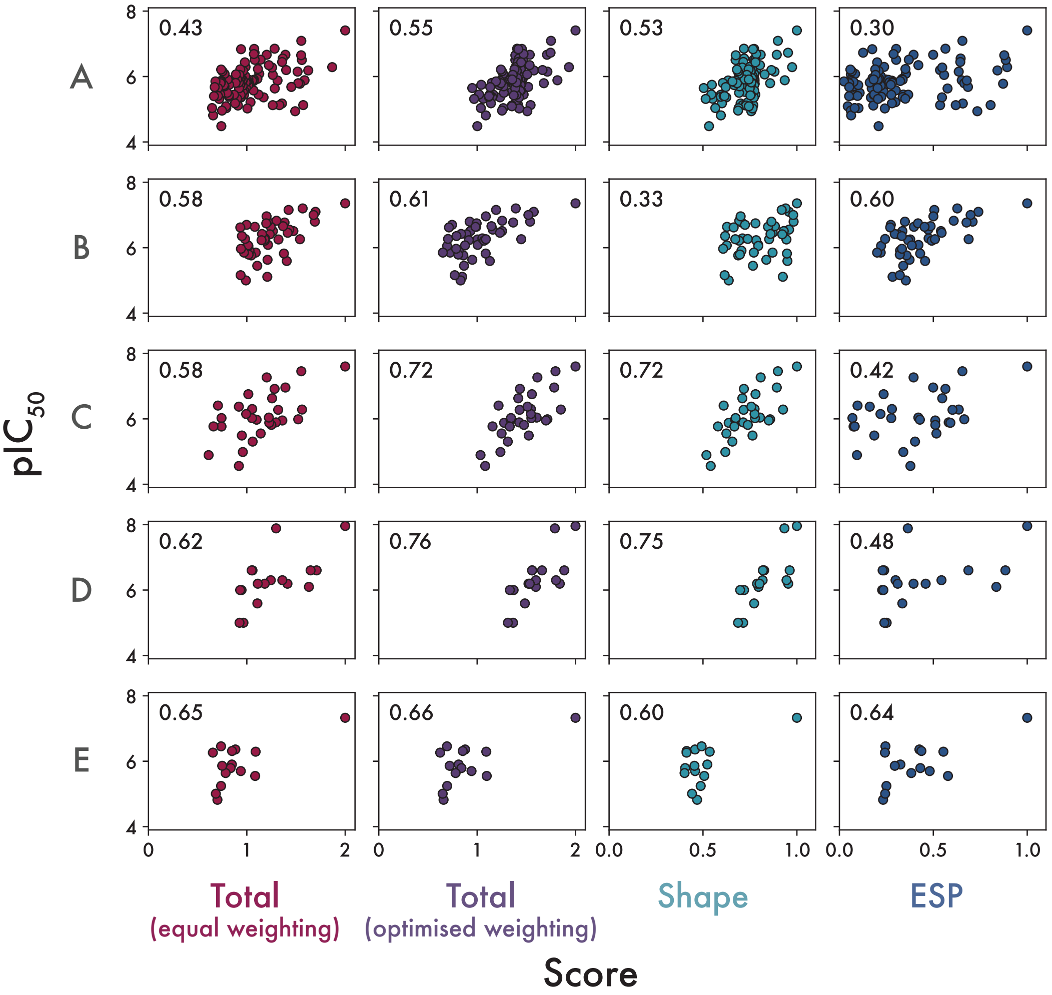

Figure S11: The NLRP3 series correlations, before and after optimising the ESP and shape weightings in the total score. The Pearson correlation coefficient is shown in the top left-hand corner.

Cross-validation

To assess whether the series-specific weightings obtained from retrospective optimisation reflect genuine structure–activity trends rather than in-series overfitting, we performed repeated out-of-sample validation within each matched molecular series. For each series, the dataset was randomly partitioned into training (80%) and test (20%) subsets. Shape and electrostatic similarity weights were fitted on the training subset only using the same constrained SLSQP optimisation procedure

described in the main text, maximising the Pearson correlation between the weighted similarity score and pIC_{50}. The fitted weights were then applied without modification to the held-out test subset, and Pearson and Spearman rank correlation coefficients between weighted similarity and pIC_{50} were evaluated. This procedure was repeated 50 times with different random splits, and results are reported as the mean ± standard deviation of the held-out correlations and fitted weights. Owing to the limited size of series D and E, substantial variance in held-out correlations is expected; the purpose of this analysis is to assess robustness of the retrospective weighting rather than to establish prospective predictive performance.

Table S9: Repeated train/test validation of optimised shape and electrostatic similarity weighting for the NLRP3 matched molecular series. For each series, results are reported as mean ± standard deviation over 50 random splits.

Series		Pearson Coefficient		CV weighting	
	n	In-sample	Held-out	Shape	ESP
A	105	0.55	0.50 ± 0.15	0.90 ± 0.04	0.10 ± 0.04
B	46	0.61	0.61 ± 0.19	0.19 ± 0.09	0.81 ± 0.09
C	30	0.72	0.69 ± 0.25	0.97 ± 0.04	0.03 ± 0.04
D	17	0.76	0.54 ± 0.55	0.91 ± 0.07	0.09 ± 0.07
E	15	0.66	0.35 ± 0.54	0.41 ± 0.27	0.59 ± 0.27

S-8 3,5-Disubstituted pyrazolo[1,5-a]pyrimidine SwissBioisostere Results

These are the known bioisosteres of 3,5-pyrazolo[1,5-a]pyrimidine retrieved from a SwissBioisostere search on 28 December 2024, and are displayed in Table S10. The nomenclature in the title line is taken directly from SwissBioisostere, with the frequency being the total number of times that this MMP appears in their dataset, 'better' being the number of times this substitution improved bioactivity, 'similar' being a retention of bioactivity, and 'worse' a deterioration in bioactivity.

Table S10: The results of a SwissBioisostere search of 3,5-pyrazolo[1,5-a]pyrimidine.

Known Bioisostere SMILES	Frequency	Better	Similar	Worse
[*:1]c1cnc2ccc([*:2])nn12	33	15	15	3
[*:1]c1nnc2ccc([*:2])cn12	14	1	3	10
[*:1]c1ccc2cnc([*:2])nn12	13	1	8	4
[*:1]c1ccc2ccc([*:2])nn12	9	0	5	4
[*:1]c1csc2ncc([*:2])nc12	9	0	8	1
[*:1]c1cnn2ccc([*:2])cc12	5	2	3	0
[*:1]c1cnc2ccc([*:2])cn12	5	0	3	2
[*:1]n1cnc2ccc([*:2])nc12	5	0	2	3
[*:1]c1ccc2c([*:2])ncnn12	4	0	2	2
[*:1]c1c([*:2])nn2cccnc12	3	1	1	1
[*:1]c1nnc2ccc([*:2])nn12	3	1	2	0
[*:1]n1cnc2cnc([*:2])cc12	3	1	2	0
[*:1]n1cnc2ccc([*:2])cc12	3	0	3	0
[*:1]c1cnn2cc(O[*:2])cnc12	2	1	0	1
[*:1]c1cnn2cc([*:2])cnc12	2	1	0	1
Cc1cc([*:2])n2ncc([*:1])c2n1	2	0	1	1
Cc1cc([*:2])nc2c([*:1])cnn12	2	0	1	1
Cc1nc2ccc([*:2])nn2c1[*:1]	2	0	2	0
Cc1nn2ccc([*:2])nc2c1[*:1]	2	0	2	0
[*:1]n1nnc2ccc([*:2])nc12	2	0	0	2
[*:1]c1cnn2ccc(NCCC[*:2])nc12	1	1	0	0
[*:1]c1cnn2ccc(NCC[*:2])nc12	1	1	0	0
[*:1]c1ncc2ccc([*:2])cn12	1	1	0	0
CN([*:1])c1nnc2ccc([*:2])cn12	1	0	1	0
CN([*:2])c1ccn2ncc([*:1])c2n1	1	0	1	0
COCc1cc([*:2])n2ncc([*:1])c2n1	1	0	1	0
COCc1cc([*:2])nc2c([*:1])cnn12	1	0	1	0
COc1cc2nnc([*:1])n2cc1[*:2]	1	0	1	0
Cc1cc(CO[*:2])n2ncc([*:1])c2n1	1	0	1	0
Cc1cc(CO[*:2])nc2c([*:1])cnn12	1	0	1	0
Cc1cc([*:2])nn2c([*:1])nnc12	1	0	0	1
Cc1cc2nnc([*:1])n2nc1[*:2]	1	0	0	1
FC(F)(F)c1cc([*:2])nc2c([*:1])cnn12	1	0	1	0
FC(F)(F)c1nnc2cc(O[*:1])c([*:2])cn12	1	0	1	0
FC(F)c1cc([*:2])nc2c([*:1])cnn12	1	0	1	0
Nc1cc([*:2])nc2c([*:1])cnn12	1	0	1	0
[*:1]Cc1nnc2ccc([*:2])nn12	1	0	0	1
[*:1]c1cccc([*:2])n1	1	0	0	1
[*:1]c1cn2cc([*:2])ccc2n1	1	0	0	1
[*:1]c1cnc2ccc(OC[*:2])nn12	1	0	1	0
[*:1]c1cnc2ccc(O[*:2])nn12	1	0	1	0
[*:1]c1cnn2c(C[*:2])ccnc12	1	0	1	0
[*:1]c1cnn2c([*:2])ccnc12	1	0	1	0
[*:1]c1cnn2ccc(O[*:2])nc12	1	0	1	0

S-9 VEHICLe Sample for Geometry RMSD Benchmarking

Table S11: The RegIDs of the sample of MoBiVic used for geometry, charge, and conformer benchmarking.

S550759	S384174	S240734	S838949	S497018	S172238	S484588	S30726
S576937	S215804	S277806	S510075	S630110	S444642	S266201	S28911
S454146	S445146	S830015	S253389	S113960	S670290	S659282	S737081
S545164	S484122	S184798	S641387	S444933	S152689	S384206	S472050
S602717	S630380	S365278	S770274	S812007	S654747	S372069	S322354
S773674	S369317	S56525	S26286	S425220	S831956	S171132	S838165
S130539	S222990	S39786	S445966	S636686	S694961	S739371	S563707
S575605	S76423	S737334	S796428	S782642	S666848	S90053	S687860
S67472	S517006	S63038	S178302	S49591	S8617	S340289	S537730
S370806	S600986	S369022	S634214	S265310	S52843	S723400	S395589
S661848	S290126	S528861	S237261	S550098	S265196	S191093	S501140
S121934	S240303	S658147	S221830	S695380	S587245	S310891	S181415
S442223	S261798	S441571	S227218	S625601	S528809	S277234	S125855
S456932	S529107	S550463	S252683	S832821	S523702	S666146	S68550
S59578	S112068	S26431	S564684	S753422	S545055	S624513	S141323
S237351	S408594	S411925	S567178	S291068	S831893	S527705	S472822
S563499	S741989	S172380	S17939	S813395	S689519	S782678	S238841
S551486	S288105	S821119	S818810	S540371	S800360	S826635	S248365
S428058	S294945	S785613	S407508	S307042	S60410	S425441	S395184
S271807	S55494	S374767	S768461	S762646	S554718	S529793	S760163
S840313	S576270	S466068	S474392	S729123	S791624	S731066	S442622
S113628	S425162	S630466	S550900	S381945	S326222	S448120	S67221
S732414	S547205	S753201	S841988	S453251	S331042	S675554	S577961
S793088	S46691	S78003	S552241	S539706	S695035	S602885	S18366
S1117	S780763	S71463	S152902	S179942	S369299	S277014	S301862
S408382	S722552	S116370	S505398	S94145	S139559	S142433	S677276
S226136	S657558	S444762	S628242	S418971	S488375	S248168	
S453689	S632488	S551366	S566157	S311787	S62245	S220686	
S518946	S568236	S773109	S476323	S217543	S500098	S720207	
S185618	S579503	S455825	S90133	S371724	S215775	S718691	
S763490	S219118	S373290	S711293	S243640	S733587	S512238	
S365681	S499866	S560414	S521829	S678807	S369310	S501575	

S-10 Molecular Context Benchmarking

The HCIE methodology treats the user-specified query heterocycle and the library heterocycles as fragments, isolated from the chemical context of any parent molecule. We wished to investigate the effects that this widely-made assumption in ligand-based virtual screening would have on the HCIE search results. To partially test this assumption, we took the 95 071 returned heterocycles from the HCIE search for pyrazolo[1,5-a]pyrimidine illustrated in Figure 6 and constructed repotrectinib analogues for each heterocycle by joining them to the repotrectinib parent scaffold at the defined attachment points. Each analogue then underwent a geometry optimisation and Gasteiger partial charge calculation, using the same methods as used in HCIE. The coordinates and charges of each heterocycle, in the context of the parent molecule, were then extracted into multimolecule XYZ files and charge files respectively. This produced a library of heterocycles with information about the molecular context of the parent analogue, with the shape and ESP descriptors reflecting the influence of the whole molecule. The pyrazolo[1,5-a]pyrimidine query heterocycle was treated in the same way, and searched using the HCIE two-vector alignment and scoring algorithm against this 'context-aware' library. This process is summarised in Figure S12.

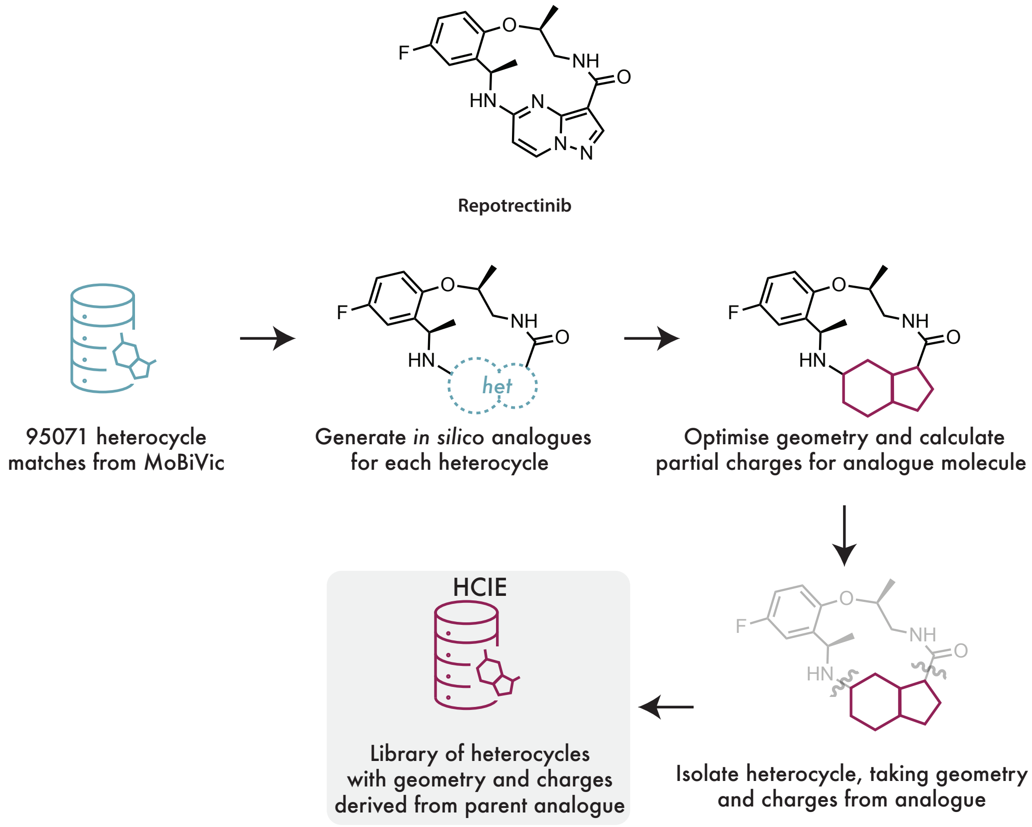

Figure S12: Building a 'context-aware' library for HCIE searching by calculating the key ESP and shape descriptors for each heterocycle in the context of the parent molecular scaffold.

In the contextualised search, eight out of the top ten molecules shown in Figure 6 were present in

the top ten scoring results[a], and within the top 1000 molecules were 618 of the original heterocycles. These results suggest that the fragment-only approximation captures much, but not all, of the context-dependent effects relevant to the local shape and ESP descriptors. For prioritising candidates and exploring heterocyclic chemical space, we believe that these common assumptions give sufficiently reliable results, however should users wish to incorporate contextual information into their results, HCIE is written such that analyses such as these are straightforward to implement.

References

[1] Riniker, S.; Landrum, G. A. Better informed distance geometry: Using what we know to improve conformation generation. *J. Chem. Inf. Model.* **2015**, *55*, 2562–2574.

[2] Tosco, P.; Stiefl, N.; Landrum, G. Bringing the MMFF force field to the RDKit: implementation and validation. *J. Cheminform.* **2014**, *6*, 37.

[3] Kim, S.; Bolton, E. E.; Bryant, S. H. PubChem3D: conformer ensemble accuracy. *J. Cheminform.* **2013**, *5*, 1.

[4] Gasteiger, J.; Marsili, M. Iterative partial equalization of orbital electronegativity—a rapid access to atomic charges. *Tetrahedron* **1980**, *36*, 3219–3228.

[5] Bayly, C. I.; Cieplak, P.; Cornell, W.; Kollman, P. A. A well-behaved electrostatic potential based method using charge restraints for deriving atomic charges: the RESP model. *J. Phys. Chem.* **1993**, *97*, 10269–10280.

[6] Cornell, W. D.; Cieplak, P.; Bayly, C. I.; Kollman, P. A. Application of RESP charges to calculate conformational energies, hydrogen bond energies, and free energies of solvation. *J. Am. Chem. Soc.* **1993**, *115*, 9620–9631.

[7] Jakalian, A.; Bush, B. L.; Jack, D. B.; Bayly, C. I. Fast, efficient generation of high-quality atomic charges. AM1-BCC model: I. Method. *J. Comput. Chem.* **2000**, *21*, 132–146.

[8] Jakalian, A.; Jack, D. B.; Bayly, C. I. Fast, efficient generation of high-quality atomic charges. AM1-BCC model: II. Parameterization and validation. *J. Comput. Chem.* **2002**, *23*, 1623–1641.

[9] Frisch, M. J. et al. Gaussian16 Revision C.01. 2016; Gaussian Inc. Wallingford CT.

[10] Case, D. A. et al. AmberTools. *J. Chem. Inf. Model.* **2023**, *63*, 6183–6191, PMID: 37805934.

[a] Because several heterocycles had identical total scores, they were assigned equal rank. As a result, the set of molecules within the "top ten scores" for the contextualised results contains more than ten heterocycles.
